# Causes and consequences of crossing-over evidenced via a high-resolution recombinational landscape of the honey bee

**DOI:** 10.1186/s13059-014-0566-0

**Published:** 2015-01-02

**Authors:** Haoxuan Liu, Xiaohui Zhang, Ju Huang, Jian-Qun Chen, Dacheng Tian, Laurence D Hurst, Sihai Yang

**Affiliations:** State Key Laboratory of Pharmaceutical Biotechnology, School of Life Sciences, Nanjing University, Nanjing, 210093 China; Department of Biology and Biochemistry, University of Bath, Bath, BA2 7AY UK

## Abstract

**Background:**

Social hymenoptera, the honey bee (*Apis mellifera*) in particular, have ultra-high crossover rates and a large degree of intra-genomic variation in crossover rates. Aligned with haploid genomics of males, this makes them a potential model for examining the causes and consequences of crossing over. To address why social insects have such high crossing-over rates and the consequences of this, we constructed a high-resolution recombination atlas by sequencing 55 individuals from three colonies with an average marker density of 314 bp/marker.

**Results:**

We find crossing over to be especially high in proximity to genes upregulated in worker brains, but see no evidence for a coupling with immune-related functioning. We detect only a low rate of non-crossover gene conversion, contrary to current evidence. This is in striking contrast to the ultrahigh crossing-over rate, almost double that previously estimated from lower resolution data. We robustly recover the predicted intragenomic correlations between crossing over and both population level diversity and GC content, which could be best explained as indirect and direct consequences of crossing over, respectively.

**Conclusions:**

Our data are consistent with the view that diversification of worker behavior, but not immune function, is a driver of the high crossing-over rate in bees. While we see both high diversity and high GC content associated with high crossing-over rates, our estimate of the low non-crossover rate demonstrates that high non-crossover rates are not a necessary consequence of high recombination rates.

**Electronic supplementary material:**

The online version of this article (doi:10.1186/s13059-014-0566-0) contains supplementary material, which is available to authorized users.

## Background

**Figure 1:**
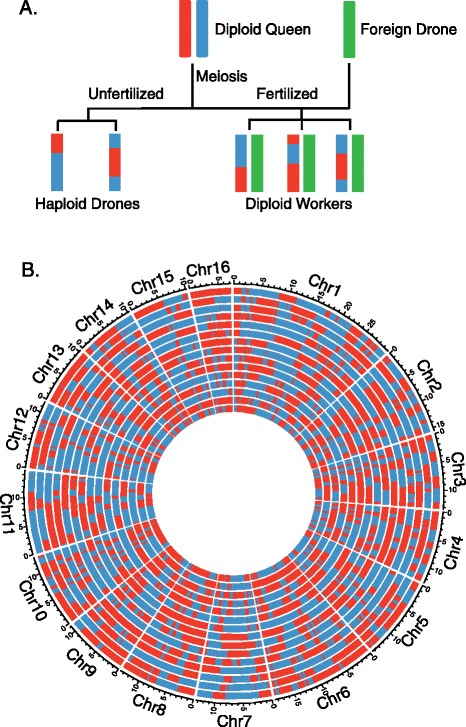
**Relationship of queen-drone-worker and recombination map of drones.** **(A)** Schematic description of the queen-drone-worker relationship within a colony; **(B)** recombination map of the 15 drones in colony II. Each circle represents one drone, the samples from outmost to innermost are: II-5; II-6; II-7; II-8; II-16; II-17; II-23; II-24; II-26; II-27; II-28; II-32; II-35; II-36; II-38.

To understand the causes and consequences of crossing over, ideally one would study a species with easy to resolve recombination, high intragenomic variation in recombination rates and high mean rates. Social hymenoptera, especially the honey bee (*Apis mellifera*), are in this context strong candidates for a model species. Numerous studies have shown that social hymenoptera have the highest recombination rate among animals studied to date [1-3]. The honeybee (*Apis mellifera*), in particular, has the highest crossing-over rate (19 cM/Mb) in any animal or plant, estimated from approximately 3,000 genetic markers along one-third of the genome [4]. The recombination rate in honey bees is also highly variable around the genome with both acute hot and cold spots of recombination [4]. The underlying haploid-diploid genetics of hymenoptera also holds rare advantages for analysis. A honeybee colony is headed by a single queen and includes dozens of drones and thousands of workers [5]. The haploid drones develop from unfertilized eggs, while workers develop from fertilized eggs and hence are diploid [6] (Figure 1A). The haploid nature of the drones obviates difficulties associated with heterozygosity, making inference of recombination relatively straightforward (effectively equivalent to sperm typing). This combined with their diploid queen in the same colony [6] make for good material to study meiotic recombination.

Here we make use of these advantages and derive a high-density recombination map of the honey bee genome. We employ two sets of queen-drone combinations and one queen-drone-worker combination. These were sequenced with high coverage (approximately 36× on average). In our study, approximately 700,000 accurate markers were employed to call the recombination activity across the whole genome at a fine scale in each colony (mean interval between markers 314 bp). We use the resource to address a series of questions regarding the causes and consequences of crossing over.

Our recombination map is of sufficiently high resolution to potentially detect both crossing-over events and the finer scale gene conversion events. There are two ways to resolve a double strand break (DSB) during meiosis, crossover (CO) and non-crossover (NCO) [7]. Meiotic crossover leads to the exchange of homologous chromosomes and yields new allelic combinations at a large scale, normally involving 500 kb or longer sequences [8]. The same DSB can, however, be resolved in a manner that does not involve crossing over, so called NCO events [8-11]. These events involve gene conversion that can alter a small piece of DNA, usually less than 2 kb, from one haplotype chromosome to another [12,13]. Gene conversion events are also associated with crossover events. Indeed, in yeast about 60% to 70% of gene conversions are associated with crossing over [8,14] and more than half of all crossovers have associated gene conversions [8,14]. One disadvantage of using haploid males (rather than tetrad analysis) is that we are likely to underestimate the rate of crossover-associated gene conversion (see Methods).

### The causes of the ultra-high crossing-over rate in social hymenoptera

While the causes of the high recombination rate in social insects remain unresolved, a few possibilities have been excluded [2,3], notably that: (1) it is not a *de facto* consequence of haplodiploidy as the asocial *Nasonia* has much lower rates at 1.4 to 1.5 cM/Mb [15]; and (2) it is not simply owing to domestication, as undomesticated social wasps [2] and ants [16] also have high rates. Moreover there is no evidence for the typical population genetical domestication fingerprints in honey bee, this possibly owing to the frequent admixture of the managed honey bee [17]. By elimination, the high crossing-over rate appears to be a property of eusociality. We examine two hypotheses, what may be called the immune-function hypothesis and the worker diversification hypothesis, both of which propose a coupling between eusociality and high crossing-over rates. The premise of the tests of these hypotheses is that selection for a given function should in turn be able to explain which genes have unusually high crossing-over rates in their proximity [18,19].

The immune-function hypothesis supposes a connection with increased immune demands of social species [20]. Social species may be particularly vulnerable to infectious disease owing to among other things: (1) physical proximity, making transmission easier; (2) close relatedness, ensuring there to be many vulnerable individuals in close proximity; and (3) because of increased temperatures associated with social species [21,22]. Such an explanation for increased crossover rates is attractive given the large body of evidence suggesting a potential coupling between the evolution of sex and recombination and host-parasite co-evolution [23]. However, rather paradoxically social insects appear to be losing immune genes [24-26] and those that remain appear to be under relaxed constraint rather than positive selection [27]. Nonetheless, we address the immune-crossover hypothesis by asking whether the recombination rate in the vicinity of immune-related genes is in any manner unusual.

An alternative hypothesis for the high recombination rates in social hymenoptera holds that the frequent meiotic recombination may contribute to the evolution of behavior of workers, which may provide the primary driving force to allow social insects to adapt to their environment [18,19]. While the precise logic of the argument has been configured in a variety of ways [28,29], a claimed prediction of this hypothesis is that crossing over should be more common in the vicinity of genes that act in worker brains [18,19]. Assuming a correlation (possibly owing to biased gene conversion (GC)) between local GC content and the CO rate [4], a recent study [18] found some indirect support for this possibility, showing that genes with biased expression in the brains of workers also have higher GC content. The team thus drew the inference that crossing over was associated with the evolution of worker behavior due to the strong links between these two factors in honeybees [18].

Not only is direct evidence of a link between crossing over and worker-brain gene expression still lacking, the facts and interpretation are far from clear. If the NCO gene conversion rate is high as claimed [30], the correlation between GC content and worker-brain gene expression could arise as a result of NCO events were these also associated with biased GC. Note, however, in yeast biased GC is associated exclusively with CO-associated gene conversion [31]. Possibly more problematically, Hunt *et al.* have noted [32] that genes with queen-biased expression also have high GC content, thus questioning whether worker genes are in any manner unique.

This latter issue, we suggest, may fit within a broader context. In humans genes that are more broadly expressed (that is, expressed in many tissues) tend to have low local recombination rates, while tissue specific genes tend to be recombinogenic [33]. While the cause of this correlation is unknown, it suggests a general antagonism between gene expression (possibly in the germ line) and crossing over. Given that genes that are biased in expression in any manner (queen biased, brain biased, and so on), will by definition sit closer towards the tissue-specific end of the spectrum, any correlation between brain expression and crossing over may, in line with Hunt *et al.*’s [32] objection, simply be owing to a more general correlation between breadth of expression and crossing over. If so, there would be no good reason to suppose that the recombination data in any manner support the view that crossing over in honey bees is related to selection for worker diversification. We return to this issue asking if the crossing-over rate near genes upregulated in worker brains is in any manner unusually high and whether, if this is the case, this can be explained as a side consequence of covariates.

### The consequences of crossing over

The second set of issues that we wish to resolve concern the consequences of crossing over. The first possible consequence of high CO rates that we wish to understand concerns the relationship between two modes of resolving DSBs during meiosis. Given these two major means (CO and NCO) to resolve a DSB and given an unusually high CO rate in honey bees, does it follow that the NCO rate in honey bees is unusually low or might it be that increased CO rates is accompanied by increased NCO rates? Conceptually we can postulate three scenarios: (1) selection for increased recombination was on the total number of DSBs, with the relative proportion resolved one way or the other unchanged; or (2) selection alters the proportion of DSBs resolved one way rather than the other, leaving total DSB counts largely unaltered; or (3) selection is on other minor pathways to resolve DSBs, like non-homologous end joining or sister chromatid recombination or restored gene conversions, that cannot be detected in our study [7,11,34]. Naturally, a hybrid model is also viable.

The direct estimation of gene conversion rate at a genome-wide scale is difficult owing to the small size of gene conversion spans, with relatively few markers in the converted events [8,35,36]. In a recent study, an oocyte method was employed to detect the recombined pairs of SNP site from the genome sequences obtained from a mixed pool of haploid males [30]. They concluded that the honeybee has about 30 times more gene conversions than CO events [30] suggesting a very high NCO rate. This provides no support for the second of the three scenarios above, this being at the upper reaches of gene conversion-crossover ratios seen across taxa. It also suggests that restored gene conversions (that is, gene conversions that leave no footprint as they do not affect sequence) are unlikely to be common. However, this study could not distinguish the copy number variations in the genome, which can cause the non-allelic sequence alignments and lead to false positive calling of gene conversion events [36,37]. Therefore, they might overestimate the number of gene conversions. With our high-resolution landscape we consider it worthwhile to return to this issue.

Some consequences of recombination are thought to be direct effects [38]. Most notably, in many taxa we see a correlation between recombination rates and GC content [4,39,40]. The dominant explanation for this is that it reflects the biased repair of heteroduplex mismatches (meiotic intermediaries) favoring GC residues over AT residues [11]. However, as noted above, whether any correlation is due to gene conversion during crossing over or owing to gene conversion during NCO recombination events (for example, during synthesis dependent strand annealing) is important to resolve, not least because it is now commonplace to presume that the local GC content can be employed to infer the local crossing-over rate. If most gene conversion is via NCO events, and NCO events are also associated with biased gene conversion (although this appears not to be so in yeast [31]), such an assumption would be questionable.

A third predicted consequence that we wish to test for concerns the degree of diversity held in the genomes within the population. Because of effects of linkage, mutations of selective effects can interfere (affect the fate) of those in linkage disequilibrium with them [41]. A consequence of such interference (for example, as factored in Hill-Roberston interference [42]) is that the physical span of interference should be lower when the local crossing-over rate, per Mb, is higher. The effect of this should be to enable increased diversity in domains of high recombination, all else being equal. Prior analyses of the bee genome failed to report a significant trend [4] while a recent study, based on population genetic estimates of crossover rates, has found a significant relationship between divergence and crossing-over rate [18]. Given that the trend has not been reported from a direct estimate in all taxa [38], and given the centrality of this issue within population genetics, this issue is worth returning to.

## Results

### Marker identification and haplotype phasing

Three bee colonies, I, II, and III, were sampled from hundreds of colonies in the same farm. Fifty-five individuals, including three queens (one from each colony), 18 drones from colony I, 15 drones from colony II, 13 drones and six workers from colony III, were used for whole-genome sequencing. After sequencing, 43 drones and six workers were resolved to be offspring of their corresponding queens, whereas three drones from colony I were identified with a foreign origin. In excess of 150,000 SNPs were shared by these three drones but could not be detected in their corresponding queen (Figure S1 in Additional file 1). These drones were removed for further analysis. The diploid queens were sequenced at approximately 67× depth, haploid drones at approximately 35× depth, and workers at approximately 30× depth for each sample (Table S1 in Additional file 2).

To ensure the accuracy of the called markers in each colony, four strategies were employed (see Methods for details): (1) only these heterozygous single nucleotide polymorphisms (hetSNPs) called in queens can be used as candidate markers, and all small indels are ignored; (2) to exclude the possibility of copy number variations (CNVs) confusing recombination assignment these candidate markers must be ‘homozygous’ in drones, all ‘heterozygous’ markers detected in drones being discarded; (3) for each marker site, only two nucleotide types (A/T/G/C) can be called both in the queen and drone genomes, and these two nucleotide phases must be consistent between the queen and the drones; (4) the candidate markers must be called with high sequence quality (≥30). In total, 671,690, 740,763, and 687,464 reliable markers were called from colonies I, II, and III, respectively (Table S2 in Additional file 2; Additional file 3).

The second of these filters appears to be especially important. Non-allelic sequence alignments caused by copy number variation or unknown translocations can lead to false positive calling of CO and gene conversion events [36,37]. Because drones from the same colony are the haploid progenies of a diploid queen, it is efficient to detect and remove the regions with copy number variations by detecting the hetSNPs in these drones’ sequences (Tables S2 and S3 in Additional file 2; see methods for details). A total of 169,805, 167,575, and 172,383 hetSNPs, covering approximately 13.1%, 13.9%, and 13.8% of the genome, were detected and discarded from colonies I, II, and III, respectively (Table S3 in Additional file 2).

To evaluate the accuracy of the markers that passed our filters, three drones randomly selected from colony I were sequenced twice independently, including independent library construction (Table S1 in Additional file 2). In principle, an accurate (or true) marker is expected to be called in both rounds of sequencing, because the sequences are from the same drone. When a marker is present in only one round of the sequencing, this marker might be false. By comparing these two rounds of sequencings, only 10 out of the 671,674 called markers in each drone were detected to be different due to the mapping errors of reads, suggesting that the called markers are reliable. The heterozygosity (number of nucleotide differences per site) are approximately 0.34%, 0.37%, and 0.34% between the two haplotypes within colonies I, II, and III, respectively, when assessed using these reliable markers. The average divergence is approximately 0.37% (nucleotide diversity (π) defined by Nei and Li [43] among the six haplotypes derived from the three colonies) with 60% to 67% of different markers between each two of the three colonies, suggesting each colony is independent of the other two (Figure S1 in Additional file 1).

In each colony, by comparing the linkage of these markers across all drones, we can phase them into haplotypes at the chromosome level (see Figure S2 in Additional file 1 and Methods for details). Briefly, when the nucleotide phases of two adjacent markers are linked in most drones of a colony, these two markers are assumed to be linked in the queen, reflective of the low-probability of recombination between them [44]. Using this criterion, two sets of chromosome haplotypes are phased. This strategy is highly effective in general as in nearly all locations there is only one recombination event, hence all drones bar one have one of two haplotypes (Figure S3 in Additional file 1). A few regions are harder to phase owing to the presence of large gaps of unknown size in the reference genome, a feature that leads to a large number of recombination events occurring between two well described bases (see Methods). In downstream analyses we ignored these gap containing sites unless otherwise noted.

### The consequences of high crossing-over rates

#### Honey bees have very high crossover rates and low non-crossover rates

With the phased haplotypes of chromosomes of the queens, we could identify recombination events in each drone [35]. In each colony, we get mosaic drone chromosomes with genotype switching from one haplotype to the other of the queen (Figure 1B; Figure S2B and Figure S4 in Additional file 1), which might be the result of COs or gene conversions. After filtering these potential non-allelic sequence alignments, the genotype switching points were detected along the chromosomes to identify the CO or gene conversion events. Since almost all directly observed gene conversions in other taxa have tract lengths considerably less than 10 kb [8,45], we assume that the spans with >10 kb are an outcome of COs. If spans less than 10 kb with identical genotype derived from one of the two haplotypes of the queen are assumed to be the outcome of gene conversions (including crossover-associated gene conversions and non-crossover gene conversions), while spans >10 kb are presumed to be COs, a total of 3,505 COs and 250 gene conversion events were detected in the 43 drones (these include the sites of multiple COs associated with large gaps, Additional file 4). Of these 250 gene conversions the majority (221) are not in proximity to CO events and indicate, we assume, NCO events. Given a genome of size 220 Mb (combined length of assembled chromosomes), with an average of 81.5 COs per genome, we estimate a CO rate of 37 cM/Mb and 5 to 6 NCO gene conversions per drone per meiosis (Table 1 and Table S4 in Additional file 2). NCO events in gap regions could not be detected while CO events in gap regions in principle can sometimes be detected. Given a 9.04% gap in the genome, the actual number of NCOs would be 9.04% higher, this being a minor correction.

**Table 1:** **Numbers of crossover and gene conversion events in each colony**

	**Colony**	**Crossover events (Track length)**			**Gene conversions**
		**>500 kb**	**>100 kb**	**>10 kb (cM/Mb)**	**≤10 kb**
Drone	I	48.5	72.6	82.1 (37)	7.2
	II	52.8	74.1	85.0 (39)	4.7
	III	50.5	70.5	76.8 (35)	5.5
Workers^a^	III	48.8	72.0	82.0 (37)	^b^

^a^Six workers come from colony III.

^b^Gene conversions in workers were not identified.

Blocks with span >10 kb are counted as crossovers and ≤10 kb are counted as gene conversions.

We note that relaxing the 10 kb assumption for the span to define whether a recombination tract should be defined as gene conversion or crossover makes little to no difference (Additional file 1: Figure S5). If we impose an upper limit for gene conversions of 1 kb then the number of gene conversions (both NCO and CO-associated) goes down a tiny bit and the CO rate goes up (to 4.86 and 83.42, respectively); if we suppose gene conversion tracts can be up to 20 kb the comparable numbers resolve to 6.21 versus 80.72. Thus the finding that most recombination events are crossovers and NCO gene conversion appears to be rare is robust to our 10 kb assumption. This largely reflects that rarity of recombination events in the 1 to 20 kb range, as expected if gene conversion is rare and tracts are short. Moreover even if we are ‘more generous’ to gene conversion events, increasing the cutoff value to 100 kb, we recover only 10.3 gene conversions per drone and 72.5 crossovers per drone. As our method should be robust to calculating NCO gene conversion rates, the above figures, which capture both NCO and rare complex CO associated gene conversion, are liberal estimates of the NCO gene conversion rate (given constraints imposed by marker density). An apparently low NCO gene conversion rate thus appears to be a robust conclusion.

An implicit assumption we make is that the recombination rate measured in drones is reflective of that experienced by genes transmitted to workers. As meiosis occurs before worker/drone specification, *a priori* we expect that genes in workers and drones to have experienced the same recombination rate. This is indeed the case. We find sampling six workers from colony III (Methods for details) that the number of crossover events in each haplotype (82.0 ± 8.6, in the range of 69 to 90; Table S1 in Additional file 2) is no different from that witnessed in drones (two-tailed Brunner-Munzel test, *P* = 0.90).

These crossover per Mb estimates come with some uncertainty given the lack of assurance about the genome size and the size of the gaps associated with the domains where we observe multiple recombination events between two well described markers. Even if we remove all the instances in which we observe more than one recombination event between the same two markers, despite the mapping and phasing around these breakpoints being good, the CO rate drops to 52 crossover/drone (24.5 cM/Mb). And if we remove shared COs that happened in five or more drones, the CO rate drops to 68 crossover/drone (31.3 cM/Mb). We are inclined to suppose that the higher estimates may be the more accurate if only because the estimate of the total genome size is probably quite accurate. In removing multi-crossovers associated with gaps we remove the COs and the annotated gap size from the calculation. However, the real length of these gaps is uncertain and each of these gaps is represented by a run of 50,000 Ns. When we remove shared COs, cM drops severely but Mb drops only a little, which may simply reflect the fact that the gap sizes are mis-stated. We have 3,505 COs in total, 2,245 are identified in only one drone, the rest (100*2 + 80*3 + 59*4 + 50*5 + 30*6 + 22*7 = 1,260) are identified in ≥2 drones, so when we remove all the shared COs about one-third of all COs are removed.

No matter which estimate we employ, the CO rate estimated in this study is higher than that previously estimated [4]. This we hypothesized may be owing to the higher marker density and more complete genomic information in this study (average 314 bp interval between two adjacent markers) than Beye’s study (average approximately 100 kb interval). To address this we randomly picked a certain number of markers to reconstruct a recombination map. Net recombination rate is relatively tolerant to removal of quite a few markers but plummets when marker density goes too low (Figure S6 in Additional file 1). These simulations suggest that with circa 300 evenly scattered markers we would estimate a recombination rate around 19 cM/Mb (the original estimate). Whether this captures the prior analysis is, however, unclear as that analysis examined scaffolds covering only one-third of the genome. Nonetheless, a difference between analyses is expected given our higher density and more complete genome build.

**Figure 2:**
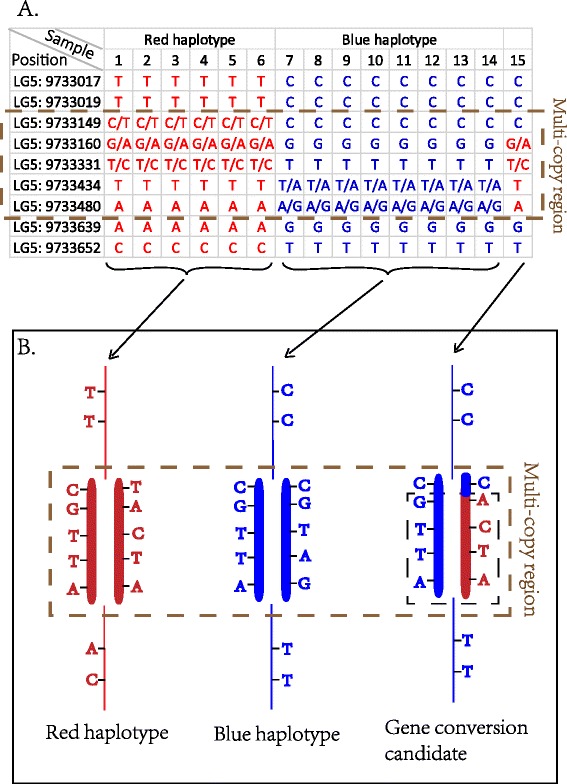
**Schematic representation of recombination events in multi-copy regions.** Multi-copy region is marked by brown dotted frame and gene conversion event is marked by black dotted frame. **(A)** Genotypes of 15 samples around a multi-copy region on chromosome 5. A gene conversion event is identified in sample 15. **(B)** Sketch illustration of inferred red haplotype, blue haplotype, and haplotype with gene conversion event.

Theoretically, aside from CNVs, sequencing errors, or mapping errors, hetSNPs are unexpected in the genome of haploid drones but make up about 13% of the genome. Notably, most of such hetSNPs distribute in clusters, suggesting copy number variation as the underlying cause (Figure 2). If the genotype changes in these regions can be assumed to be fairly reported then these could provide a unique opportunity to identify gene conversion candidates in multi-copy regions. However, this assumption may well not be safe. Nonetheless, they afford the opportunity to test whether our low estimated gene conversion rate is due to the discarded regions with drone-hetSNPs. To this end we explored the gene conversions in these drone-hetSNP regions, even though these gene conversions may experience a higher false positive risk. In some of the multi-copy regions, we can discriminate between the two haplotypes (as shown in Figure 2A, red and blue represent two haplotypes), if a drone’s genotype changes from one type to another, a potential gene conversion is identified (Figure 2 and Figure S7 in Additional file 1; see Methods for details). Counting all of these potential gene conversion events, only 45 candidates were detected in the copy number variation regions in the 43 drones (Figure 2 and Figure S7 in Additional file 1, and Table S5 in Additional file 2). When adding these gene conversions, only 6.8 gene conversions are observed per drone per meiosis. This is significantly lower than the recent estimation that the honeybee has about 30 times higher gene conversions than the number of CO events [30].

#### The problem of the missing crossover-associated gene conversion events

At first sight, the majority of gene conversions appear to be associated with NCO events. When we define a CO-associated gene conversion as one within 10 kb of a CO event, of the 250 gene conversions 29 are CO-associated (approximately 12% of all gene conversions). If we permit the critical distance to go to a probably unrealistic 100 kb this figure resolves to 43, with the remaining 207 being NCO gene conversions. This suggests that the majority (circa 80% or above) of gene conversions are not associated with crossing over. These data also suggest that a very minor fraction (29/3505 = 0.8%, 43/3505 = 1.2%) of CO events are associated with gene conversions, which is in striking contrast to what is observed in yeast where the majority of CO events have associated gene conversions [8,14].

We caution strongly against interpreting the above results as they stand. While in yeast, for example, it is possible to recover tetrads, in bees this is not possible. As a consequence, we may miss many simple gene conversion events associated with COs, for such events may merge the conversion event with the CO event and hence will be classified as a single CO event when viewed in a single haploid (see Methods). Complex conversion events by contrast are expected to leave the trace we think we can discern. We see no reason why this issue should affect estimation of the NCO rate. Thus our inference of the CO-associated gene conversion rate is most likely an underestimate.

Evidence from yeast suggests that the underestimation may be acute as the majority (90%) of CO-associated recombination events are of the simple variety [8]. If we assume the same proportion in bees this suggests that we may be missing 261 of 290 cross-over associated gene conversion events and that more realistic estimate for the total number of gene conversions per drone is circa 12 (290 CO-associated gene conversion events of which 10%, 29, are complex and discernable, and 221 NCO events = 511 gene conversion events across 43 drones, approximately 12 per drone). If these figures are correct, it still suggests that only about 8% of crossing-over events have an associated gene conversion tract, still much lower than in yeast. However, this result by necessity is sensitive to assumptions about the relative rate of complex and simple gene conversions associated with crossing over. If, for example, we are missing 99% of CO-associated gene conversion events then we could be missing circa 3,000 events and the majority of CO events have a gene conversion event. The haploid drone system does not readily permit estimate of the rate of simple versus complex events so we leave undecided the number of CO-associated gene conversions.

#### Distribution of the recombination events along the genome

**Figure 3:**
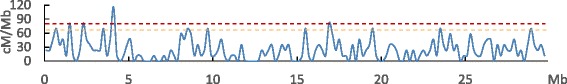
**Recombination rate variation along chromosome 1.** Rate above the red dotted line is CO hotspot for *P* <0.01 and rate above the yellow dotted line is CO hotspot for *P* <0.05.

The abundant recombination events in honeybees distribute highly unevenly along the chromosomes (Additional file 5). The recombination rate varies between 0 and 197 cM/Mb when measured in non-overlapping 200 kb windows across chromosomes (Figure 3, Figure S8 in Additional file 1 and Table S6 in Additional file 2). A total of 58 CO hot-regions (Poisson distribution, *P* <0.05) locating at approximately 10 Mb regions were identified, and 54 CO cold regions (Poisson distribution, *P* <0.05), with a combined length of 31.2 Mb, were detected. In other words, approximately 25% of CO events are clustered within approximately 5% of the whole genome (Table S7 in Additional file 2), and approximately 14% of the genome is entirely devoid of CO events (Table S6 in Additional file 2). Chromosome 1 had the largest number of recombination hot regions (12 out of 54; Table S6 in Additional file 2). However, the domains with the highest recombination rate (197.7 cM/Mb) were observed on chromosomes 2 (Chr2: 6,200,000 to 6,400,000) and 6 (Chr6: 5,600,000 to 5,800,000), this rate being approximately 5.3-fold higher than the genome average. Even in some high recombination regions, many COs and gene conversions were found to cluster within some very small regions (for example, <10 kb).

Chromosome physical length is strongly correlated with the number of CO events per chromosome (*r* = 0.95, *P* <10^-4^; Figure S9 in Additional file 1). This suggests that the number of events per unit physical distance is approximately a constant. Indeed, as then expected, chromosome length is not correlated with the CO rates per Mb (*P* = 0.21; Figure S9D in Additional file 1). Though the recombination rate variation between chromosomes is less dramatic (36 ± 6.1 cM/Mb on average, in the range of 27 to 45), relatively higher CO rates were observed on chromosomes 1, 3, 4, and 10 (44.1 cM/Mb on average) than that on chromosomes 9, 11, and 15 (26.9 cM/Mb on average) (Table 2).

**Table 2:** **Recombination rate of each chromosome (including multi-crossovers)**

	**Length (Mb)**	**Average number of crossovers**	**Recombination rate (cM/Mb)**
Chr1	29.9	13.1	43.9
Chr2	15.5	5.9	38.0
Chr3	13.2	5.9	44.7
Chr4	12.7	5.5	43.6
Chr5	14.4	4.7	32.8
Chr6	18.5	6.9	37.1
Chr7	13.2	4.9	37.0
Chr8	13.5	4.3	31.9
Chr9	11.1	3.0	26.8
Chr10	13.0	5.7	44.0
Chr11	14.7	4.0	27.1
Chr12	11.9	4.6	38.5
Chr13	10.3	4.1	39.7
Chr14	10.3	3.6	35.0
Chr15	10.2	2.7	26.9
Chr16	7.2	2.6	35.9
Total	219.6	81.5	37.1 (Average)

#### Crossing over is associated with GC content, nucleotide diversity, gene density, and CNVs

Previous studies have shown that the recombination rate has a weak positive correlation with GC-content in 125 to 250 kb sequence windows in the honeybee [4], possibly owing the GC-biased gene conversion. Do we find the same and are breakpoints associated with higher GC content as expected if CO breakpoints are where CO-associated gene conversion is acting?

**Figure 4:**
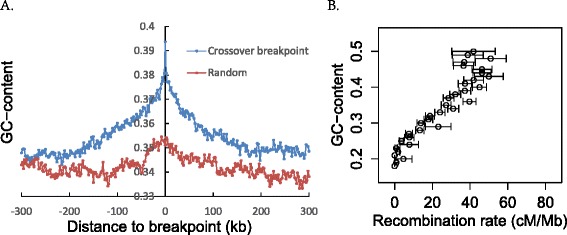
**Relationship between recombination and GC-content. (A)** GC content variance around CO breakpoints (blue dots and line). The window 0 on the x-axis is the GC content of the breakpoints and the negative and positive values represent the distance away from the breakpoints. Each of these windows is defined as 2 kb sequence and the GC content is calculated for each window. The red dots and line are one of the GC content random samples simulated like the numbers of CO breakpoints (blue dot and line). After 10,000 repeats, not one of random samples is as extreme as the observed (blue line) (*P* <0.0001). **(B)** Relationship between recombination and GC content. When the chromosomes are dissected into 10 kb non-overlapping regions, recombination rate (cM/Mb) and GC content can be obtained for each of them. After the bins are sorted by the GC content, the windows are divided into 31 groups based on GC content (approximately 20% to 51%, 1% interval), and the average (and s.e.m.) recombination rates reported for each group.

As regards the second issue, we indeed find that the breakpoint regions have higher GC content than their surrounding regions and that the closely surrounding regions have higher GC-content than the genome average or the randomly simulated data (Figure 4A and Figure S10 in Additional file 1).

To ask about the relationship between GC content and recombination rate we employ two approaches. In both we dissect the genome into 10 kb non-overlapping windows of which there are 19,297. First, we ask about the raw correlation between GC% and cM/Mb for these windows, which as expected is positive and significant (Spearman’s rho = 0.192; *P* <10^-15^). Second, we wish to know the average effect of increasing one unit in either parameter on the other. Given the noise in the data (and given that current recombination rate need not imply the ancestral recombination rate) we approach this issue using a smoothing approach. We start by rank ordering all windows by GC content and then dividing them into blocks of 1% GC range, after excluding windows with more than 10% ‘N’. The resulting plot is highly skewed by bins with very high GC (55% to 58%) as these have very few data points (Additional file 1: Figure S10E) (the same outliers likely effect the raw correlation too). Removing these three results in a more consistent trend (Additional file 1: Figure S10F). This also suggests that below circa 20% GC the recombination rate is zero (Additional file 1: Figure S10F). Removing those with GC <20% and, more generally, any bins with fewer than 100 windows (all bins with GC < 20% have fewer than 100 windows) leaves 18,680 (96.8%) of the windows, these having a GC content between approximately 20% and 51%. These are used to construct Figure 4B, which presents a relatively noise-free (after smoothing) monotonic relationship between the two variables.

By observation, we estimate that on average a 1 cm/Mb increase in recombination rate is associated with an increase in GC content of approximately 0.5%. Conversely a 1% increase in GC content corresponds to an approximately 2 cM/Mb increase in recombination rate. We conclude that given the apparent rarity of NCO gene conversion, at least in the bee genome, extrapolation from GC content to average crossing-over rate thus seems to be justifiable, at least for GC content over 20%. We note too that at the extreme GC contents the recombination rate may be over or underestimated. This may reflect a discordance between current and past recombination rates.

Crossing-over rate is also associated with nucleotide diversity, gene density, and copy number variation regions (Figure S11-S13 in Additional file 1) [46]. Given our removal of hetSNPs from analysis the latter result is not trivially a CNV associated artifact. Our fine-scale analyses reveal a positive correlation between nucleotide diversity and recombination rate at all the scales of 10, 100, 200, or 500 kb sequence windows (Figure S11 in Additional file 1). This bolsters prior analyses, one of which [4] reported the trend but found it to be non-significant, while another [18] reported a trend between population genetic estimates of recombination and genetic diversity. The trend accords with the notion that recombination causes reduced Hill-Robertson interference thus enabling reduced rates of hitchhiking and background selection, so enabling greater diversity. We also find a strong negative correlation between recombination and gene density (Figure S12 in Additional file 1) and a strong positive correlation between recombination and the length of multi-copy regions at various window sizes (Figure S13 in Additional file 1). The correlation with CNVs is consistent with a role for non-allelic recombination generating duplications and deletions via unequal crossing over [47].

#### No robust evidence for motif enrichment near crossovers

We further analyzed the importance of specific GC-rich motifs that previously have been shown to influence recombination rate [48,49]. These included CpG, (CCT)n, CCTCCCC, CCTCCCT, and CCTCCCCT. All were found to be enriched in the breakpoint regions and highly correlated with the local GC-content. As such an enrichment compared with randomly selected genomic sequences may be an artifact of heightened local GC content, we also ask whether shuffled versions of the motifs are enriched (given that CpG is just two nucleotides with the negative strand identical to the positive strand the analysis here is not possible). To this end, we shuffle each motif 10,000 times and for each variant we ask how commonly we see it in the real sequence. If there are M incidences of the real variant being as common or more common that the shuffled variant then *P* = (M + 1)/10,001. Except for motifs of (CCT)_3_ (*P* = 0.012) and (CCT)_4_ (*P* = 0.002) we observe that original versions of other motifs are no more enriched in the genome than are their shuffled versions: (CCT)_2_, *P* = 0.268; CCTCCCC, *P* = 0.278; CCTCCCT, *P* = 0.468; CCTCCCCT, *P* = 0.215. We note that the level of significance for the (CCT)_3_ is not robust to multitest correction. We note too that the two significant motifs are trinucleotide repeats of the same motif and hence their abundance, relative to shuffled versions, may be explained by whatever causes repeat expansions, rather than any relationship with crossing over *per se*.

### The causes of high crossing-over rates

#### Worker brain expression predicts crossing-over rates

A prior claim, based on GC content, identified that recombination rates are highest in the vicinity of genes with expression in worker brains [18]. This in turn was suggested to relate to the debate concerning the causes of the exceptionally high recombination rate particular to social hymenoptera [3]. Can we also confirm whether an association with brain/behavior predicts crossover rates? As genes with drone-fat body-biased expression might be associated with the male courtship behavior, as seen in *Drosophila* [50], we include these genes in the set of behavior-related genes. Using the worker-, drone-, and queen-biased expression genes [51,52] to associate with CO events, we could define sets of genes showing biased expression, that is to say highly upregulated in a given tissue compared to some comparator.

**Figure 5:**
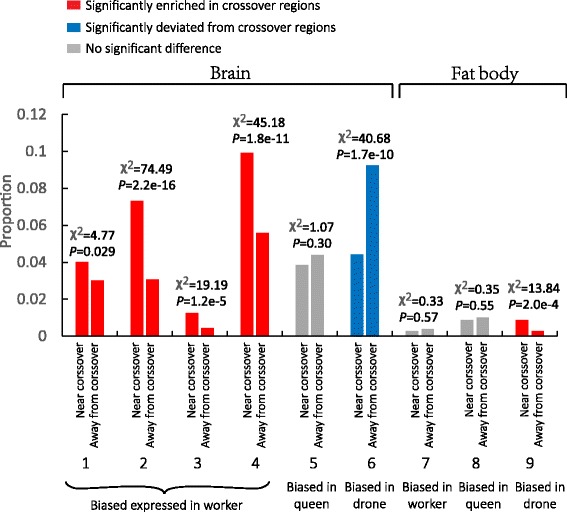
**Relationship between genes with biased expression and recombination regions.** The horizontal line represents nine sets of genes; genes in each set were divided into two groups, near crossover or away from crossover, as annotated below the horizontal line. The statistics were performed by Chi-square test with a 2 × 2 table comparing between whole-genomic genes and the nine sets of genes. Each vertical bar represents the proportion of genes in each set compared with whole-genomic genes. Genes significantly enriched in crossover regions are marked in red, genes significantly deviated from crossover regions are marked in blue, and genes showing no significance are marked in grey. The nine sets of genes are: 1, biased expressed genes in worker’s brain [51]; 2, biased expressed genes in worker’s brain [52]; 3, genes overlapped in the first two sets; 4, union set of genes in the first two sets; 5, biased expressed genes in queen’s brain [51]; 6, biased expressed genes in drone’s brain [52]; 7, biased expressed genes in worker’s fat body [53]; 8, biased expressed genes in queen’s fat body [53]; 9, biased expressed genes in drone’s fat body [53].

Comparing the local (within 10 kb) crossover rates of genes that have worker-brain-biased expression compared with expression in queen brains, we observe a weak enrichment in the vicinity of COs, not significant after Bonferonni testing (*P* = 0.029 before multiple testing). Worker-brain biased genes defined via upregulation compared with drone-brain biased genes, show a much greater enrichment in the vicinity of COs (*P* = 2.2 × 10^-16^). Both the overlapping set and the union set of these former two classes also show robust enrichment near COs (Figure 5). Conversely genes expressed preferentially in drone brains compared with worker’s brain show evidence of avoidance of crossovers (*P* = 1.7 × 10^-10^; Table S8A in Additional file 2). We find no trend as regards queen-biased expression genes compared with worker’s brain between, around and away from the breakpoint regions (*P* = 0.3; Figure 5, Table S8A in Additional file 2). These results largely confirm the suggestion of Kent *et al.* [18] that worker-brain-biased genes are unusual in having high crossover rates. Drone-fat body-biased expression genes compared with worker’s fat body also show a robust enrichment within or/and around the breakpoint regions of the COs (*P* = 2.2 × 10^-16^; Figure 5, Table S8A and D in Additional file 2), suggesting that other behavior-related genes might also be implicated.

Given the correlation noted above between high gene density and low crossing-over rates, perhaps the association with worker brains simply reflects their residing in domains of low density? The gene density across the whole genome is 4.1 genes/100 kb. Surprisingly, then worker-brain-biased genes reside in regions of higher gene density, averaging 9.0 genes/100 kb, and so, all else being equal, should have low crossing-over rates. Indeed, when compared with other genes from domains of similar gene density, worker-brain bias genes are very highly enriched near COs (chi sq-test, *P* <2.2e-16: 173/579 for worker-brain-bias genes vs. 69/516 for genes in similar gene density). The correspondence with high gene density is not simply a consequence of tandem gene duplication favoring worker-brain-biased genes. Of 752 worker genes 701 are single copy genes (query and hit with overlapping region >50% and identity >50% are treated as paralogs). These singletons are closer (<10 kb) to recombination hotspots than expected by chance (*P* <1e-9 in chi square-test, method as in Table S8) and in domains of high density (9 genes/100 kb). These results provide *prima facie* support for the hypothesis that selection favors the higher recombination rate in worker brain genes.

Similar results were also observed in regions with copy number variations, such that the genes with worker-brain biased expression compared with queen’s or drone’s brain are strongly associated with the copy number variations (Table S8B and S8C in Additional file 2). By contrast the drone-brain-biased expression genes (defined in comparison with worker’s brain) are significantly absent from these regions (Table S8B and S8C in Additional file 2). This is perhaps to be as expected as the CNV regions are likely to also be the result of recombination (via unequal crossover [47]). Most of these multi-copy regions should be the result of unequal CO because these multi-copies seems to be tandem duplications in the genome inferred from the close distance (1 kb-5 kb) between the paired-end reads.

A coupling between crossing over and brain expression is also suggested by analysis of genes with well-described functions. At the breakpoint hotspot regions of COs, there are 42 well-annotated genes, whose function had been verified in the honeybee or fruit fly. Interestingly, 17 of them, including six worker-brain-biased expression genes, have functions in the nervous system or behavior (Table S9 in Additional file 2).

Interpretation of the above results may yet be problematic. In the human genome, a striking negative correlation between within-gene CO rate and expression breadth has been observed [33]. Might it be that a biased expression in worker brains simply is indicative of greater tissue specificity and thus high crossing-over rates? After analyzing the EST data and protein atlas in organs and tissues of bees, we fail to detect any trend relating expression breadth to CO rates (spearman rho = -0.036, *P* = 0.12, N = 1,874). It might be that germline expression in queens is what matters, if so breadth of expression in queens might be the key variable. However, breadth of expression in queens is also unrelated to the CO rate: rho = -0.073, *P* = 0.28, N = 1,727. We thus fail to detect any relationship between CO rates and tissue specificity.

#### No evidence that immune genes have unusual crossing-over rate

Another suggestion for high recombination rates suggests strong parasite driven pressure [20]. In 150 immune-related genes annotated in Evans’ study [24], commonly genes in Toll, Imd, and JAK/STAT pathways, 27 of them are found near a CO breakpoint (distance ≤ 10 kb). This is not significantly different to what would be expected for a random gene (Chi squared *P* >0.05).

#### Sex determination genes are in a recombinational desert

In addition to the above, we can also ask whether certain types of gene are associated with CO deserts or hotspots. We employ Gene Ontology (GO) analysis to ask which gene classes show the highest crossing-over rates. This reveals that G protein-coupled receptor (*GPCR*) genes, which have been confirmed to sense signals outside the cell and mediate cellular response and are crucial for an organism’s behavior in response to environment [54], are most enriched within the CO breakpoint regions (Table S10 in Additional file 2). Intriguingly, a recent study has found signs of common positive selection in GPCR genes of honey bee [55]. As the rate of gene conversion associated with CO events is uncertain, we cannot exclude the possibility that this coupling is owing to biased gene conversion giving artifactual signals of positive selection.

One domain stands out as a recombinational desert. No CO or gene conversion event was detected in an approximately 400 kb region surrounding the two linked sex determination genes, *csd* and *fem* (Additional file 1: Figure S14). We suggest that, as heterozygosity of *csd* determines the sex of honeybee [56,57] and diploid homozygotes are sterile males, gene conversion within this domain would be disadvantageous. As gene conversion is associated both with CO and NCO events, all recombination should be abolished to avoid homogenization. An absence of recombination also forces the two loci to behave as a single haplotype.

Our result stands in striking contrast to an earlier report [58] that found exceptionally high CO rates in the vicinity of the *csd* locus. A possible resolution of the contradictory claims is that while in the immediate vicinity of the genes there is no recombination, this might be counterbalanced by unusually high rates in the spans proximal to the desert. A lower resolution analysis would detect the higher rates in the spans. Consistent with this we observe a high peak of recombination in the immediate vicinity of the plateau (Additional file 1: Figure S14).

## Discussion

### The highest crossover rate observed in animals and plants

Our data add to the notion that social hymenoptera have both high and highly variable crossing-over rates. Indeed, the rate varied between 0 and 197 cM/Mb when measured in non-overlapping 200 kb windows across chromosomes (Figure 3, Figure S8 in Additional file 1 and Table S6 in Additional file 2), suggesting the highly uneven distribution of crossovers in the genome. The new figure of 37 cM/Mb, while high for animals, is still below that seen for some fungi and protozoans, where rates in excess of 60 cM/Mb are reported [3]. To the best of our knowledge, the new estimate suggests a higher crossing-over rate than seen in any plant or animal.

While our study finds evidence consistent with several theoretical predictions, cause and effect are always hard to disentangle from correlation alone. For example, in principle the diversity/crossing-over coupling is also consistent with the notion that crossing over occurs preferentially in domains of high diversity and with the notion that crossing over is mutagenic [59]. Similarly, the correlation between recombination rate and GC content is consistent with both the possibility that crossing over forces a high GC content and with the possibility that a high GC content favors increased crossing over. The conventional wisdom holds that recombination forces heteroduplexes within which mismatches require resolution. If this resolution is biased towards G and C residues (biased gene conversion), then a correlation between GC and recombination rate is expected [11]. Previous analyses in honey bee have demonstrated fixation biases toward GC in high recombination parts of the genome [18]. Evidence to support this direction for the causal arrow requires SNP analysis showing a bias to AT- > GC SNPs being fixed (or otherwise favored) compared to GC- > AT SNPs at sites of recombination. The key events here are most probably crossover-associated gene conversion events, but for these we cannot determine the direction of conversion and we are likely to be missing the majority of them. In our identified NCO events, if *u* is the number of AT → GC SNPs per A or T and *v* is the number of GC → AT SNPs per G or C, then the ratio of *u/v* is 1.06, which is slightly greater than the null (from stochastic simulations with 10,000 repeats: *P* <0.08). Being on the edge of significance, we cannot robustly say that NCO is or is not associated with GC-biased gene conversion.

### A low NCO gene conversion rate

A recent report suggests that the honeybee has about 30 times higher gene conversions than the number of CO events by analysis of the panel of raw reads from the DNA admixture of haploid drones [30]. This suggests that an increase in the CO rate was also associated with an increase in the gene conversion NCO rate. However, as mentioned above, there is on average approximately 13% of the genome that resides in CNV regions in the honeybee. Unfortunately such regions could not be resolved in Bessoltane’s study [30] and these multi-copy numbers will result in non-allelic sequence alignments and could lead to false positives for gene conversions [37]. Therefore, it is possible that Bessoltane *et al.* [30] may have overestimated the number of gene conversion events.

Our data suggest that on average only six to seven gene conversions per meiosis can be detected in the honeybee, of which five or six are NCO events, which appears to be one of the lowest gene conversion rates recorded in higher eukaryotes (cf human [10] and yeast [8]). While our estimate is slightly lower than that in *Drosophila* (approximately 13 per meiosis) [9] comparison between species is not simple as the ability to resolve gene conversions is highly dependent on both marker density and method. Indeed, with the uncertainty over the missing simple CO-associated gene conversion events, we prefer not to make any definitive statement on the total number of gene conversions. Despite these uncertainties, we cannot see how our data square with estimates of many more gene conversions than CO events [30] when we detect only five to six NCO events per meiosis and circa 80 CO events.

The low NCO rate suggests that selection to increase the CO rate has resulted in more DSBs resolved as crossovers rather than more crossovers *per se*. However, full resolution of this will require estimates of the ancestral (pre-eusociality) rates of both crossing over and gene conversion (and gene conversion resolution), estimates that are currently unavailable. Nonetheless we see no evidence for a concerted increase in both CO and NCO events, contra to what was previously suggested [30]. Thus, we conclude that higher NCO rates appear not to be a necessary consequence of, or accompaniment to, increasing CO rates.

### Recombination is strongly associated with the genes of worker behavior

Here we have provided the first direct evidence that the worker-biased brain expression genes are significantly enriched within and around the breakpoint regions of crossovers. The effect is all the more profound when comparison is made to domains of similarly high gene density. That we see no similar increase for immune-related genes strongly supports the ‘worker-eusocial brain/behavior model’ , over an increased selection on immune function model as part of the explanation for increased CO rates in eusocial taxa.

Many of the worker-brain enriched genes have known functions in the behavior or nervous system in honeybee or fruit fly. For example, the gene of *cpx* has been identified with neuronal communication function [60-62]; *mirr* mediates many activities in nervous system and is also responsible for larval escape behavior in fruit fly [63,64]; *Rgl* regulates neuroblast cortical polarity and spindle orientation and is also associated with aggressive behavior in fruit fly [65,66]; and *dunce* regulates the brain development within cAMP/CREB signaling pathways which, suggestively, is rapidly evolving in primitively eusocial bees [67-69]. By contrast, the drone-biased expression genes were significantly absent from these regions (Figure 5, Table S8 in Additional file 2), suggesting that the trends we see are not trivial correlates to brain expression *per se*.

Precisely why the CO rate is so high in social hymenoptera and in the vicinity of worker brain genes in particular is less transparent. Assuming the effect to be causal in some manner, the correlation between worker-brain expression and CO rates may reflect selection for local modifiers of the recombination rate in a zone of positive selection, to free the alleles up from selective interference [70,71]. That is to say, the modifiers of recombination are themselves the target of selection to enable positive selection. While such reduced interference is likely, whether the selection pressures are strong enough to be causal is less clear. A further possibility is that local directional selection on a quantitative trait selects for the most extreme phenotype and, by proxy, the most highly recombining individuals. This is, for example, one way to explain why domestication (commonly a form of strong directional selection) is sometimes associated with increased recombination rates [72,73]. Alternatively, there may be direct selection for variance between workers in their behavior and selection for locally high recombination rates might achieve this. In the process a high diversity at the population level will also be maintained, a diversity evidenced within our data.

## Conclusions

Here by whole genome sequencing of 55 honey bees and by constructing a high resolution recombination map in honey bee, we found that crossovers are associated with GC content, nucleotide diversity, and gene density. We also confirmed the former suggestion that genes expressed in worker brains have unusually high CO rates. Our data support the view that diversification of worker behavior, but not immune function, was a driver of the high crossing-over rate in bees. We find no evidence that the crossing-over rate is accompanied by a high NCO rate.

## Methods and materials

### Sample source, DNA extraction, and genome sequencing

Five colonies of honeybees (*Apis mellifera ligustica Spin*) were collected from a bee farm in Zhenjiang, China. Each colony contained one queen, dozens of drones, and hundreds of workers. Bees from three colonies were selected for whole genome sequencing.

The DNA of each individual was extracted using phenol/chloroform/isoamyl alcohol method. To minimize the risk of microbial contamination, the abdomens of bees were removed before DNA extraction. About 3 μg of DNA from each sample were used for whole genome resequencing while the remaining DNA was kept for PCR and Sanger sequencing. Construction of the DNA libraries and Illumina sequencing were performed at BGI-Shenzhen. In brief, paired-end sequencing libraries with insert size of 500 bp were constructed for each sample according to the manufacturer’s instructions. Then 2 × 100 bp paired-end reads were generated on IlluminaHiSEq 2000. The queens were sequenced at approximately 67× coverage on average, drones at approximately 35× coverage, and workers at approximately 30× coverage (Table S1 in Additional file 2). The sequences have been deposited in the GenBank database (accession no. SRP043350).

### SNP calling and marker identification

Honeybee reference genome was downloaded from NCBI [74]. The sequencing reads were first mapped onto reference genome with bwa [75] and then realigned with stampy [76]. Then local realignment around indels was performed by Genome Analysis Toolkit (GATK) [77], and variants were called by GATK UnifiedGenotyper.

Due to the lower accuracy of calling indel variants, only identified SNPs are used as markers. First, 920,528 to 960,246 hetSNPs were called in each queen (Table S2 in Additional file 2). Then, approximately 22% of them were removed due to the fact that these sites are also hetSNPs in at least one haploid drone (this may reflect non-allelic sequence alignments caused by CNVs, sequencing error, or low sequencing quality). Similar proportions of the hetSNPs also were observed in human sperm sequencing [10]. Finally, 671,690 to 740,763 reliable hetSNPs in each colony were used as markers to detect recombination events (Table S2 in Additional file 2).

### Haploid phasing

For each colony, the identified markers were used for haploid phasing. The linkage of every two adjacent markers was inferred to determine the two chromosome haplotypes of the queen by comparing the SNP linkage information across all drones from the same colony. Detailed methods were described in Lu’s study [44]. In brief, for each pair of adjacent hetSNPs, for example A/G and C/T, there could be two types of link in the queen ‘A-C, G-T’ or ‘A-T, G-C’. Assuming recombination events are low probability, if more ‘A-C, G-T’ drones are found than ‘A-T, G-C’ drones, then ‘A-C, G-T’ is assumed to be the correct link in the queen and vice versa. The two haplotypes can be clearly discriminated between >99% of markers (see Figure S2 in Additional file 1 for example). For linkage of the <1% markers, as shown in Additional file 1: Figure S2B, between markers at ‘LG1:20555174’ and ‘LG1:20555456’ , there are 14 ‘A-A or G-G’ type drones against 1 ‘A-G or G-A’ type drone, so ‘A-A, G-G’ is assumed to be the correct link in queen and a recombination event is identified at this site in sample I-9.

While for the vast majority of sites this method is highly robust, as most recombination events are witnessed in only one drone, in a few instances we see extraordinarily high apparently recombination rates. Indeed at approximately 10 sites per colony there are difficulties in our method because the linked markers are shared by seven or eight out of 15 drones (or six or seven out of 13 drones in colony III; Figure S15 in Additional file 1). We found that all of these sites contain in the breakpoint region large length-unknown gaps (represented by a run of 50,000 ‘N’s) in the genome. This suggests a simple explanation for the apparently high recombination frequencies at these sites, namely that the recombination rate is normal (per Mb) but as the sequence is large and missing it appears to be high when only known bases are considered. To test this we ran simulations which showed that indeed if the gaps are long enough they will introduce frequent cases like this. Nonetheless, in cases like this, we choose the linkage shared by eight drones (or seven drones in colony III) as the original linkage, this being the conservative methodology.

### Identification and classification of recombination events in drones

By comparing the genotypes of drones with the two phased haplotypes of the queen, we get mosaic drone chromosomes where genotype blocks change between two haplotypes of the queen. Block length is the physical interval between two end markers of this block. Block changing could be the result of either CO or gene conversion. As in previous studies [8,45] almost all of the tract lengths of gene conversions are considerably less than 10 kb; we consider this a safe upper bound. That is we define blocks spanning ≤10 kb as the outcome of gene conversions (including CO-associated gene conversions and NCO gene conversions), while blocks with span >10 kb are labeled as CO events. To check for robustness we also employed a variety of threshold ranges from 1 to 20 kb for CO and gene conversion (Figure S5 in Additional file 1). We also examine an upper limit of 100 kb.

To define whether gene conversion events are associated with crossing over or more likely the result of NCO events, we consider the genomic context of the conversion event. If a CO event has an associated nearby gene conversion then we expect there to be one large block (the CO event) with upstream or downstream of this, two further small blocks next to each other. That is, if 1 and 2 represent the two maternal haplotypes, the queen is:

- 1111111111111111111111111111111111111111111111
- 2222222222222222222222222222222222222222222222

and a crossover could be witnessed in a given drone as:

- 1111111111111111111111111222222222222222222222

If there is also a complex gene conversion associated with this CO event, then it could be:

- 111111111111111111111*2211*222222222222222222222

Notice then the two italicized small blocks adjacent to the larger block (see Figure S4 in Additional file 1 for a real example). If then we see two short blocks that occur next to each other and also next to a CO event in the same sample, they are considered as one CO-associated gene conversion event. Proximity in this context means that the more distant block (22) is no more than 10 kb from the edge of the CO block, that is, the span of the block marked in yellow is less than 10 kb (span is defined as the length of the block, which is the interval between two edge markers within this block). However, in cases like this, we cannot discriminate which one of the two short blocks is the converted one. All other putative gene conversion events are then called as NCO gene conversions and assumed to be the result of an NCO event (for decision tree see Figure S16 in Additional file 1). For example, assuming the physical distances are appropriate, a gene conversion far from a CO could look like this:

- 11*22*111111111111111111111222222222222222222222

This can be classified as a NCO gene conversion event. For robustness we also ask about the consequences of relaxing this 10 kb proximity assumption, allowing CO gene conversion events to be within 100 kb of a CO block.

Note that this method, as it does not include tetrad analysis, cannot easily resolve simple gene conversions associated with crossing over. For example, if a queen is:

- 1111111111111111111111111111111111111111111111
- 2222222222222222222222222222222222222222222222

CO-associated with simple gene conversion produces two chromosomes of the parental type and potentially two thus:

- 11111111111111111*2222*2222222222222222222222222
- 22222222222222222*2222*1111111111111111111111111

where the gene conversion span is italicized. Our methods, examining one haploid drone alone, would call this as one CO event and miss the simple gene conversion event. Thus haploid drone/sperm typing likely underestimates the number of gene conversions associated with crossing over.

### Identification of recombination events in workers

Six workers from colony III were also sequenced. Unlike drones, workers are diploid, one haplotype from the sequenced queen and the other from an unknown drone. To separate the queen haplotype from the foreign drone, we considered two scenarios: (1) for each marker in colony III, if this site is homozygous in the worker, then the genotype from the queen haplotype is easily determined as this homozygous one; (2) if this site is called a heterozygous SNP, and if this hetSNP (for example, A/T) is the same as the marker (for example, A/T), then we cannot decide which one is from the queen; and if this hetSNP (for example, G/T) is not the same as the marker (for example, A/T), then the one that is the same as the marker (T) is determined to come from the queen. Then we found that one main source of false positives is that hetSNP may be falsely called as homoSNP in workers, due to low read quality or uneven distribution of reads in each genotype. So gene conversion events were not identified in workers. Finally, 309,218 markers per worker are identified to map recombination events. We are reasonably confident that our methods minimize miscalling owing to CNVs in workers. In the two haplotypes of workers, one is from the queen and one from a foreign drone, and we only identify COs in the queen haplotype. When we screened for markers, the hetSNPs introduced by CNVs in this queen we detected in the drones were removed. As in addition we only analyzed COs in workers (tract length >10 kb), while most of the CNVs (>90%) we detected are <10 kb, we suggest that CNVs have minimal influence on our analysis in workers.

### Exclusion of non-allelic sequence alignments: identification of multi-copy regions and translocations

In using second-generation sequencing, detection of non-allelic sequence alignments, which can be caused by CNV or unknown translocations, is of importance, as failure to identify them can lead to false positives for both CO and gene conversion events [37].

To identify multi-copy regions we used the hetSNPs called in drones. Theoretically, the heterozygous SNPs should only be detectable in the genomes of diploid queens but not in the genomes of haploid drones. However, hetSNPs are also called in drones at approximately 22% of queen hetSNP sites (Table S2 in Additional file 2). For 80% of these sites, hetSNPs are called in at least two drones and also linked in the genome (Table S3 in Additional file 2). In addition, significantly higher read coverage was identified in the drones at these sites (Figure S17 in Additional file 1). The best explanation for these hetSNPs is that they are the result of copy number variations in the selected colonies. In this case hetSNPs emerge when reads from two or more homologous but non-identical copies are mapped onto the same position on the reference genome. Then we define a multi-copy region as one containing ≥2 consecutive hetSNPs and having every interval between linked hetSNPs ≤2 kb. In total, 16,984, 16,938, and 17,141 multi-copy regions are identified in colonies I, II, and III, respectively (Table S3 in Additional file 2). These clusters account for about 12% to 13% of the genome and distribute across the genome. Therefore, the non-allelic sequence alignments caused by CNV can be efficiently detected and removed in our study.

For the non-allelic sequence alignments caused by unknown translocations, which can lead to false positives, especially for small double CO events or gene conversions events [37], four stringent strategies were employed to exclude them: (1) if gaps in the reference genome were found within the genotype switching points of the small double CO events (block running length <1 Mb) or gene conversions, this recombination candidate was discarded due to the potential assembly errors of the reference genome; (2) allelic relationships of the converted blocks or the small double CO blocks with their genotype switching sequences (breakpoint regions) must be unambiguous in reference genomes, and events with ambiguous allelic relationships or high identity multi-copies (for example, >97% identity) were excluded; (3) for shared double crossovers and gene conversions between drones, uninterrupted mapped reads must be detected in genotype switching regions, whereas if the mapped reads were interrupted in these regions, this block was discarded due to potential translocation; (4) normal insert size (approximately 500 bp) of the pair-end reads must be detected in the switching points between the converted region and its flanking regions (including at least three unambiguous flanking markers in each side), and these blocks with abnormal insert size of the pair-end reads, for example, alignment gaps, were excluded. Through this filtering, a total of approximately 20% small double CO or gene conversion candidates were excluded due to the gaps in the reference genome or ambiguous allelic relationships.

### Confirmation of recombination events by Sanger sequencing

Thirty CO and thirty gene conversion events were randomly selected for Sanger sequencing. Four COs and six gene conversion candidates did not produce PCR results; for the remaining samples, all of them were confirmed to be replicatable by Sanger sequencing.

### Identification of recombination events in multi-copy regions

As shown in Figure S7, some of the hetSNPs in drones can also be used as markers to identify recombination events. In the multi-copy regions, one haplotype is homogenous SNP (homSNP) and the other haplotype is hetSNP, and if a SNP change from heterozygous to homogenous (or homogenous to heterozygous) in a multi-copy region, a potential gene conversion event is identified (Figure S7 in Additional file 1). For all events like this, we manually checked the read quality and mapping to make sure this region is well covered and is not mis-called or mis-aligned. As in Additional file 1: Figure S7A, in the multi-copy region of sample I-59, 3 SNPs change from heterozygous to homozygous, which could be a gene conversion event. Another possible explanation is that there has been *de novo* deletion mutation of one copy with markers of T-T-C. However, since no significant reduction of the read coverage was observed in this region, we surmise that gene conversion is more probable. As for event types in supplemental Additional file 1: Figure S7B and S7C, we also think gene conversion is the most reasonable explanation. Even though all of these candidates are identified as gene conversion events, only 45 candidates were detected in these multi-copy regions of the three colonies (Table S5 in Additional file 2).

### Data access

The sequences reported in this paper have been deposited in the GenBank database (accession no. SRP043350). For genotypes of the individuals in colonies I to III see Additional file 3 (a-c).

## Additional files

Additional file 1: Figure S1. Polygenetic relationship of the drones sequenced in this study, including the putative haplotypes of three queens. **Figure S2.** Schematic diagram of the process of haplotype phasing along the chromosome. **Figure S3.** Histogram of crossover frequency in drones for all crossover sites in the genome. **Figure S4.** Schematic diagram of crossover-associated gene conversion event. **Figure S5.** The effects of different thresholds for gene conversion and crossover on estimated rates. **Figure S6.** Plot of number of markers randomly picked against inferred recombination rate. **Figure S7.** Schematic diagram of gene conversion events in multi-copy region. **Figure S8.** Recombination rate and marker density variation along chromosomes 1 to 16. **Figure S9.** Plot of chromosome length against average number of crossovers or crossover rate on each chromosome. **Figure S10.** Relationship between recombination and GC content. **Figure S11.** Plot of nucleotide diversity between the two haplotypes against recombination rate at different scales. **Figure S12.** Plot of gene density against recombination rate in 100 kb non-overlapping sequence window. **Figure S13.** Plot of length of multi-copy region against recombination rate. **Figure S14.** Recombination rate variation around gene *fem* and *csd*. **Figure S15.** Schematic diagram of a site with seven out of 15 crossovers. **Figure S16.** Decision flow of NCO and CO discrimination in this study. **Figure S17.** Depth distribution at drone-hetSNP sites (blue) and at marker sites (red) in drones. Additional file 2: Table S1. Summary statistics for sequencing data. **Table S2.** Detected markers in three honeybee colonies. **Table S3.** HetSNPs detected in drones. **Table S4.** Number of crossovers and gene conversions in each sequenced sample. **Table S5.** Gene conversion events detected in multi-copy regions. **Table S6.** List of crossover hot- and cold-spot regions. **Table S7.** Statistics of CO hotspots in each colony. **Table S8.** Relationship between recombination regions or/and multi-copy regions and genes with biased expression in brain or fat body. **Table S9.** List of 17 genes related to nervous system or behavior. **Table S10.** DAVID annotation clusters enriched in genes overlap with crossover. Additional file 3:
**Genotype calls at marker sites of bees sequenced in this study.**
Additional file 4:
**List of crossover and gene conversion events.**
Additional file 5:
**Recombination rate variation across the genome in 10 kb windows.**

## Abbreviations

CNV: Copy number variation
CO: Crossover
DSB: Double strand break
GC: Gene conversion
GO: Gene ontology
GPCR: G protein-coupled receptor
NCO: Non-crossover
