# Supplementary material for: Causes and consequences of crossing-over evidenced via a high-resolution recombinational landscape of the honey bee
Source: Genome Biol. 2015 Jan 2;16(1):15. doi: 10.1186/s13059-014-0566-0 (PMC4305242; doi:10.1186/s13059-014-0566-0)

Figure S1. Polygenetic relationship of the drones sequenced in this study, including the putative haplotypes of three queens. The phylogenetic tree was constructed using the polymorphic sites on chromosome 1 between drones based on the bootstrap Neighbor-Joining method with number of differences model by MEGA v5.0. The 3 removed drones (Foreign1, Foreign2 and Foreign3 in the tree) found in colony I have a closer genetic relationship with colony III. The reference genome was used as an outgroup.

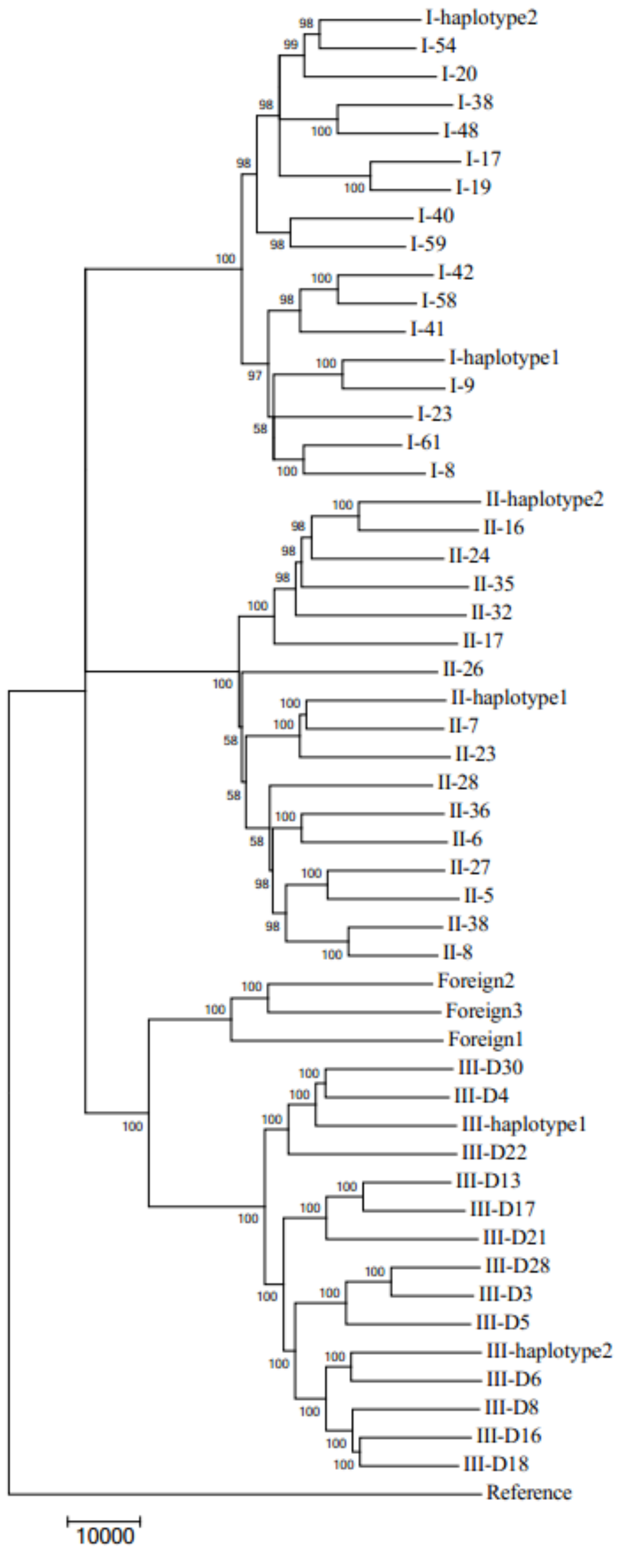

Figure S2. Schematic diagram of the process of haplotype phasing along the chromosome. A. Genotypes of 15 drones on part of chromosome 1. **B.** Genotypes of 15 drones on part of chromosome 1. A crossover event can be identified in sample I-9.

A.

Samples / Positions	Red haplotype							Blue haplotype							
	I-8	I-9	I-17	I-19	I-23	I-38	I-42	I-20	I-40	I-41	I-48	I-54	I-58	I-59	I-61
LG1:7790582	G	G	G	G	G	G	G	A	A	A	A	A	A	A	A
LG1:7790593	G	G	G	G	G	G	G	T	T	T	T	T	T	T	T
LG1:7790677	A	A	A	A	A	A	A	G	G	G	G	G	G	G	G
LG1:7790959	A	A	A	A	A	A	A	G	G	G	G	G	G	G	G
LG1:7790977	A	A	A	A	A	A	A	G	G	G	G	G	G	G	G
LG1:7791282	T	T	T	T	T	T	T	C	C	C	C	C	C	C	C
LG1:7791292	G	G	G	G	G	G	G	A	A	A	A	A	A	A	A
LG1:7791328	G	G	G	G	G	G	G	C	C	C	C	C	C	C	C
LG1:7795009	C	C	C	C	C	C	C	T	T	T	T	T	T	T	T
LG1:7798353	G	G	G	G	G	G	G	A	A	A	A	A	A	A	A
LG1:7799511	G	G	G	G	G	G	G	A	A	A	A	A	A	A	A
LG1:7799634	G	G	G	G	G	G	G	A	A	A	A	A	A	A	A
LG1:7799940	G	G	G	G	G	G	G	A	A	A	A	A	A	A	A
LG1:7800413	T	T	T	T	T	T	T	C	C	C	C	C	C	C	C
LG1:7800445	G	G	G	G	G	G	G	A	A	A	A	A	A	A	A
LG1:7800712	T	T	T	T	T	T	T	C	C	C	C	C	C	C	C
LG1:7801109	G	G	G	G	G	G	G	A	A	A	A	A	A	A	A
LG1:7801137	C	C	C	C	C	C	C	T	T	T	T	T	T	T	T
LG1:7801146	C	C	C	C	C	C	C	T	T	T	T	T	T	T	T
LG1:7801148	T	T	T	T	T	T	T	C	C	C	C	C	C	C	C

B.

Samples / Positions	Red haplotype							Blue haplotype							
	I-8	I-19	I-23	I-40	I-59	I-61	I-9	I-17	I-20	I-38	I-41	I-42	I-48	I-54	I-58
LG1:20552018	A	A	A	A	A	A	G	G	G	G	G	G	G	G	G
LG1:20552264	T	T	T	T	T	T	C	C	C	C	C	C	C	C	C
LG1:20552327	G	G	G	G	G	G	C	C	C	C	C	C	C	C	C
LG1:20552411	T	T	T	T	T	T	C	C	C	C	C	C	C	C	C
LG1:20552516	T	T	T	T	T	T	C	C	C	C	C	C	C	C	C
LG1:20553037	C	C	C	C	C	C	T	T	T	T	T	T	T	T	T
LG1:20555028	A	A	A	A	A	A	G	G	G	G	G	G	G	G	G
LG1:20555044	A	A	A	A	A	A	C	C	C	C	C	C	C	C	C
LG1:20555052	G	G	G	G	G	G	A	A	A	A	A	A	A	A	A
LG1:20555174	A	A	A	A	A	A	G	G	G	G	G	G	G	G	G
LG1:20555456	A	A	A	A	A	A	A	G	G	G	G	G	G	G	G
LG1:20555571	G	G	G	G	G	G	C	C	C	C	C	C	C	C	C
LG1:20555664	A	A	A	A	A	A	A	G	G	G	G	G	G	G	G
LG1:20555667	A	A	A	A	A	A	A	G	G	G	G	G	G	G	G
LG1:20556161	C	C	C	C	C	C	C	G	G	G	G	G	G	G	G
LG1:20556172	A	A	A	A	A	A	A	G	G	G	G	G	G	G	G
LG1:20556181	A	A	A	A	A	A	A	G	G	G	G	G	G	G	G
LG1:20556842	C	C	C	C	C	C	C	T	T	T	T	T	T	T	T
LG1:20556860	G	G	G	G	G	G	G	A	A	A	A	A	A	A	A
LG1:20556971	T	T	T	T	T	T	T	C	C	C	C	C	C	C	C

Figure S3. Histogram of crossover frequency in drones for all crossover sites in the genome. For example, the first bar indicates that for 2245 crossover sites in the genome, each crossover event is only identified in one drone. The second bar indicates that for 100 crossover sites in the genome, each crossover event is identified in two drones, and so on. We can also ask whether we observe more multiple crossovers than expected by chance. The frequency of obtaining two crossovers in the same position in the genome depends on the length of interval between two adjacent markers. If we run simulations (we use the current markers and randomly pick COs in each drone) using single crossovers alone (52 crossovers/drone), we get 48 double crossovers (two crossovers in the same genomic position) in all 3 colonies. However, if we run simulations using all the crossovers (82 crossovers/drone) we get 99 double crossovers, 13 triple crossovers and 4 quadruple crossovers in all 3 colonies. These numbers are for the most part lower than observed. However, most of the shared crossovers are located at locations with long intervals between markers, including large gap regions of unknown dimensions. As we have shown that the gaps in the genome are actually longer than reported, we expect there to be more shared crossovers than in the simulation.

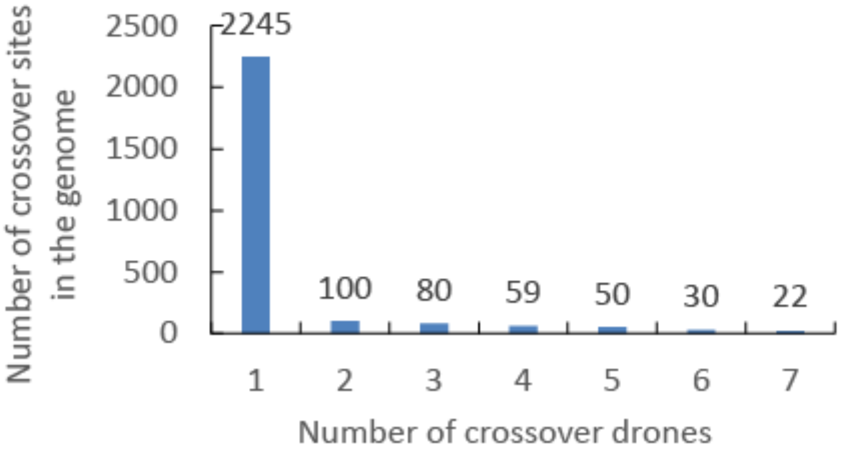

Figure S4. Schematic diagram of a crossover-associated gene conversion event. Two haplotypes of the queen are marked by red and blue, respectively. Gene conversion events are marked by black dotted frame. **A.** Two adjacent small blocks are found next to a crossover event in sample I-17, then these two blocks are identified as one crossover-associated gene conversion event; **B.** Crossover-associated gene conversion event is identified in sample II-17.

A.

Positions	Haplotype 1	Haplotype 2	Sample: I-17
LG1:23788925	T	C	C
LG1:23788974	T	C	C
LG1:23789045	C	T	T
LG1:23789085	T	C	C
LG1:23789299	A	G	G
LG1:23789324	T	C	C
LG1:23789329	A	G	G
LG1:23789332	G	T	T
LG1:23789333	A	G	G
LG1:23789412	A	G	G
LG1:23789668	G	A	A
LG1:23789803	T	C	C
LG1:23791250	C	T	C
LG1:23791289	T	C	T
LG1:23791581	C	T	C
LG1:23792930	G	A	A
LG1:23800715	A	G	A
LG1:23800867	G	A	G
LG1:23801507	C	T	C
LG1:23802728	G	A	G
LG1:23804038	A	C	A
LG1:23804184	T	C	T
LG1:23804291	A	G	A
LG1:23804997	A	T	A
LG1:23806900	G	A	G
LG1:23810415	T	C	T
LG1:23810496	C	T	C
LG1:23816399	G	A	G
LG1:23816476	A	G	A
LG1:23816541	T	G	T

B.

Positions	Haplotype 1	Haplotype 2	Sample: II-17
LG6:8968012	T	C	C
LG6:8968299	C	T	T
LG6:8968333	T	C	C
LG6:8968368	T	C	C
LG6:8969197	T	C	C
LG6:8969517	G	A	A
LG6:8969810	C	T	T
LG6:8971046	C	T	T
LG6:8975523	G	A	G
LG6:8975955	T	C	T
LG6:8975970	A	T	A
LG6:8976013	C	G	C
LG6:8976074	T	G	T
LG6:8976181	C	T	C
LG6:8977564	A	C	A
LG6:8977678	G	A	A
LG6:8977783	G	A	A
LG6:8977784	C	G	G
LG6:8977859	C	T	T
LG6:8977916	A	G	G
LG6:8978040	G	A	A
LG6:8979854	A	G	A
LG6:8979908	G	A	G
LG6:8980394	C	T	C
LG6:8980554	G	A	G
LG6:8980709	T	C	T
LG6:8980822	A	G	A
LG6:8980849	G	A	G
LG6:8980855	G	A	G
LG6:8981163	T	C	T

Figure S5. The effect of presuming different thresholds for invoking gene conversion and crossover. In the main text we assume (based on prior data from other taxa) that recombination events <10kb in span are most likely gene conversion events, not crossing over events. Here we present the modified estimation of CO and gene conversion rates upon relaxation of the 10kb threshold. The X axis presents 6 different thresholds while the Y axis presents event count. Shown in blue are inferred gene conversions per drone, in red COs per drone. Note that the absolute numbers are almost invariant.

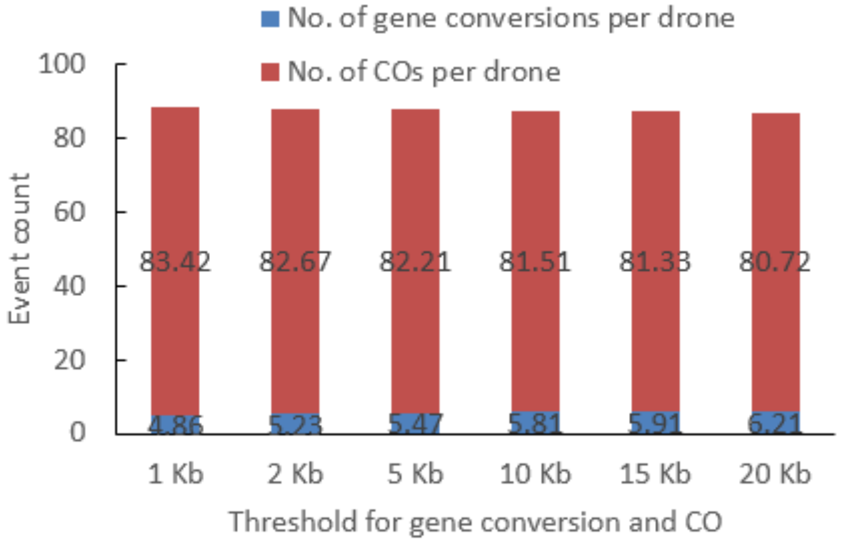

Figure S6. Plot of number of markers randomly picked against inferred recombination rate. For each point, a certain number of markers is randomly picked and a genome wide recombination rate is inferred based on the picked markers. The point estimates reflect the mean of 1000 simulations.

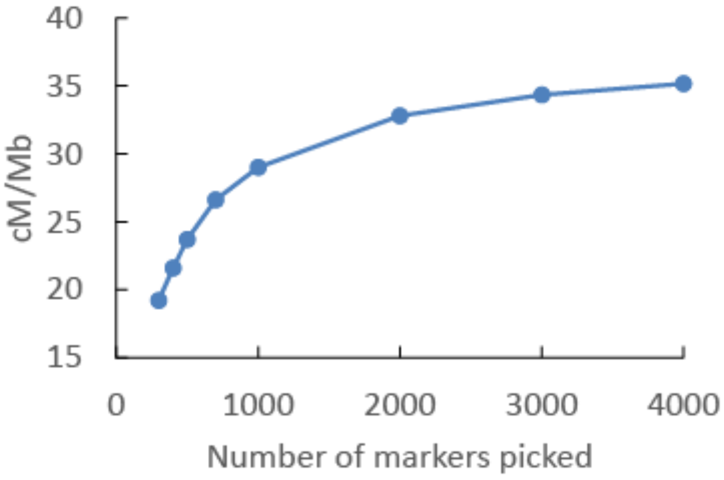

Figure S7. Schematic diagram of gene conversion events in multi-copy region. Two haplotypes of the queen are marked in red and blue, respectively. Multi-copy regions are marked by a brown dotted frame. **A.** The genotype of 3 continuous markers changes from heterozygous to homozygous (Het -> Hom) in sample I-59: a potential gene conversion event is identified accordingly. **B.** The genotype of 5 continues marker changes from homozygous to heterozygous (Hom ->Het) in sample I-54: a potential gene conversion event is identified. **C.** The genotype of 6 continuous markers changes from one haplotype to another (Het-Hom -> Het-Hom) in sample I-19: a potential gene conversion event is identified.

A. Het-> Hom

Position \ Sample	Red haplotype					Blue haplotype									
	I-20	I-23	I-40	I-41	I-61	I-8	I-9	I-17	I-19	I-38	I-42	I-48	I-54	I-58	I-59
LG15:9395300	G	G	G	G	G	A	A	A	A	A	A	A	A	A	A
LG15:9395301	A	A	A	A	A	G	G	G	G	G	G	G	G	G	G
LG15:9395335	A	A	A	A	A	T	T	T	T	T	T	T	T	T	T
LG15:9395465	C	C	C	C	C	A	A	A	A	A	A	A	A	A	A
LG15:9395483	A	A	A	A	A	G	G	G	G	G	G	G	G	G	G
LG15:9396720	C	C	C	C	C	C/T	C/T	C/T	C/T	C/T	C/T	C/T	C/T	C/T	C
LG15:9396729	C	C	C	C	C	C/T	C/T	C/T	C/T	C/T	C/T	C/T	C/T	C/T	C
LG15:9396791	T	T	T	T	T	T/C	T/C	T/C	T/C	T/C	T/C	T/C	T/C	T/C	T
LG15:9398268	G	G	G	G	G	T	T	T	T	T	T	T	T	T	T
LG15:9398409	T	T	T	T	T	C	C	C	C	C	C	C	C	C	C
LG15:9398642	G	G	G	G	G	A	A	A	A	A	A	A	A	A	A
LG15:9400647	A	A	A	A	A	G	G	G	G	G	G	G	G	G	G
LG15:9401197	A	A	A	A	A	G	G	G	G	G	G	G	G	G	G

Multi-copy region

B. Hom-> Het

Position \ Sample	Red haplotype							Blue haplotype							
	I-9	I-20	I-41	I-42	I-48	I-58	I-59	I-8	I-17	I-19	I-23	I-38	I-40	I-61	I-54
LG7:12192046	A	A	A	A	A	A	A	G	G	G	G	G	G	G	G
LG7:12192063	T	T	T	T	T	T	T	C	C	C	C	C	C	C	C
LG7:12192166	C	C	C	C	C	C	C	T	T	T	T	T	T	T	T
LG7:12192180	C	C	C	C	C	C	C	T	T	T	T	T	T	T	T
LG7:12192188	G	G	G	G	G	G	G	A	A	A	A	A	A	A	A
LG7:12192211	A/T	A/T	A/T	A/T	A/T	A/T	A/T	T	T	T	T	T	T	T	T
LG7:12192255	T/C	T/C	T/C	T/C	T/C	T/C	T/C	T	T	T	T	T	T	T	T/C
LG7:12192485	T/C	T/C	T/C	T/C	T/C	T/C	T/C	T	T	T	T	T	T	T	T/C
LG7:12192517	G/A	G/A	G/A	G/A	G/A	G/A	G/A	G	G	G	G	G	G	G	G/A
LG7:12192523	A/T	A/T	A/T	A/T	A/T	A/T	A/T	A	A	A	A	A	A	A	A/T
LG7:12192528	G/A	G/A	G/A	G/A	G/A	G/A	G/A	G	G	G	G	G	G	G	G/A
LG7:12192817	G/A	G/A	G/A	G/A	G/A	G/A	G/A	G	G	G	G	G	G	G	G
LG7:12193772	G	G	G	G	G	G	G	C	C	C	C	C	C	C	C
LG7:12193925	C	C	C	C	C	C	C	T	T	T	T	T	T	T	T
LG7:12193926	G	G	G	G	G	G	G	A	A	A	A	A	A	A	A
LG7:12193933	G	G	G	G	G	G	G	A	A	A	A	A	A	A	A
LG7:12193936	C	C	C	C	C	C	C	T	T	T	T	T	T	T	T

Multi-copy region

C. Het-Hom-> Het-Hom

Position \ Sample	Red haplotype									Blue haplotype					
	I-9	I-17	I-20	I-40	I-41	I-42	I-48	I-54	I-58	I-8	I-23	I-38	I-59	I-61	I-19
LG12:5682868	A	A	A	A	A	A	A	A	A	T	T	T	T	T	T
LG12:5682869	T	T	T	T	T	T	T	T	T	C	C	C	C	C	C
LG12:5683348	G	G	G	G	G	G	G	G	G	A	A	A	A	A	A
LG12:5683451	T	T	T	T	T	T	T	T	T	A	A	A	A	A	A
LG12:5683480	G	G	G	G	G	G	G	G	G	A	A	A	A	A	A
LG12:5687131	T	T	T	T	T	T	T	T	T	T/C	T/C	T/C	T/C	T/C	T
LG12:5687302	G/A	G/A	G/A	G/A	G/A	G/A	G/A	G/A	G/A	G	G	G	G	G	G/A
LG12:5687342	C	C	C	C	C	C	C	C	C	C/T	C/T	C/T	C/T	C/T	C
LG12:5687349	C/T	C/T	C/T	C/T	C/T	C/T	C/T	C/T	C/T	C	C	C	C	C	C/T
LG12:5687387	T/G	T/G	T/G	T/G	T/G	T/G	T/G	T/G	T/G	T	T	T	T	T	T/G
LG12:5687394	A	A	A	A	A	A	A	A	A	A/C	A/C	A/C	A/C	A/C	A
LG12:5694263	T	T	T	T	T	T	T	T	T	G	G	G	G	G	G
LG12:5694266	C	C	C	C	C	C	C	C	C	T	T	T	T	T	T
LG12:5694268	C	C	C	C	C	C	C	C	C	T	T	T	T	T	T
LG12:5694398	G	G	G	G	G	G	G	G	G	A	A	A	A	A	A
LG12:5695595	T	T	T	T	T	T	T	T	T	C	C	C	C	C	C

Multi-copy region

Figure S8. Recombination rate and marker density variation along chromosome 1~16. Recombination rate is represented by the blue line and the vertical bar on the left, marker density (10^{-4}) is represented by the red line and the vertical bar on the right. Recombination rate above the green dotted line is CO hotspot at $P<0.01$ and rate above the yellow dotted line is CO hotspot at $P<0.05$. This does not include locations with more than one crossover event.

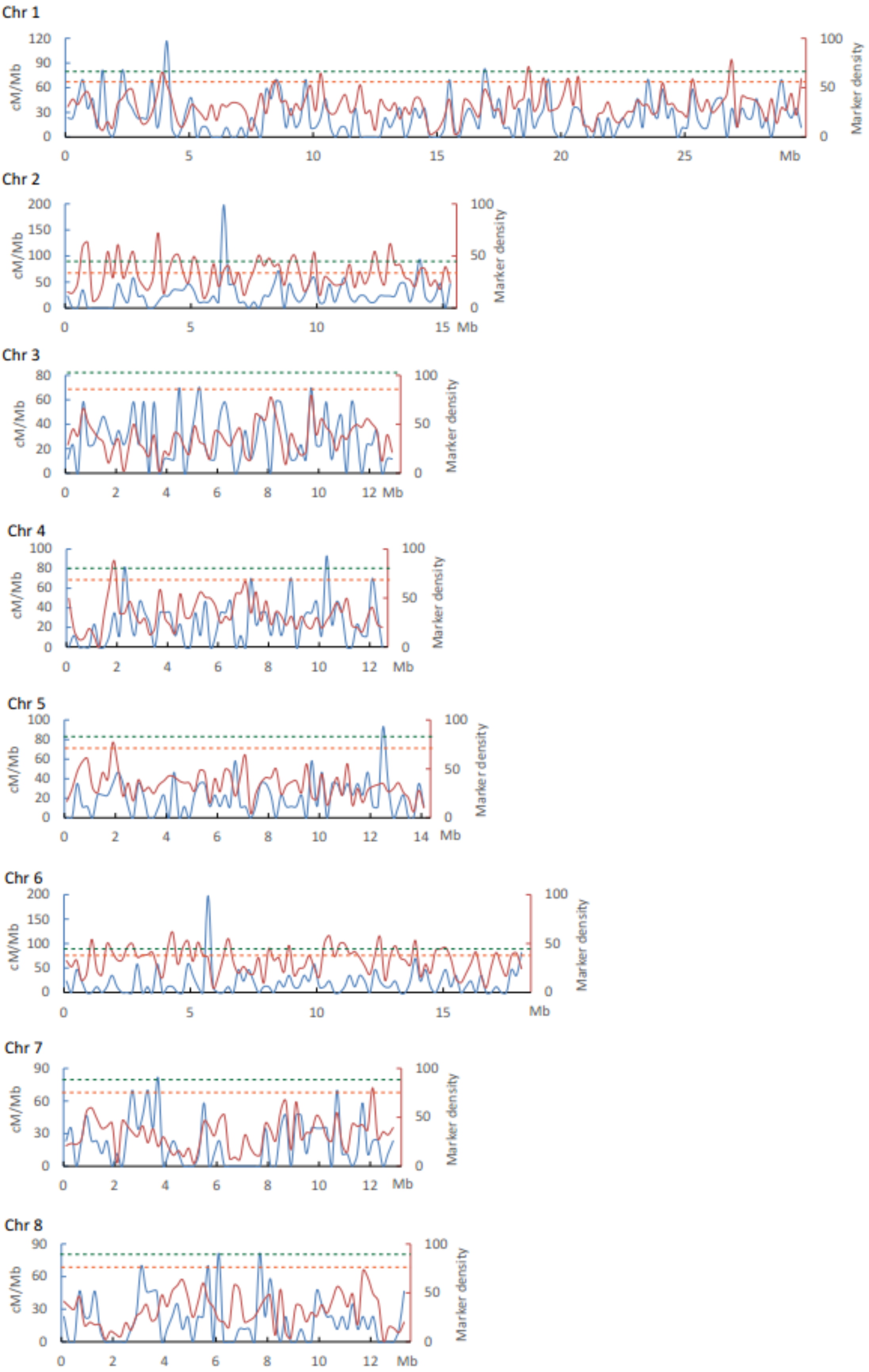

Chr 1
Chr 2
Chr 3
Chr 4
Chr 5
Chr 6
Chr 7
Chr 8
cM/Mb
Marker density
Mb

Chr 9

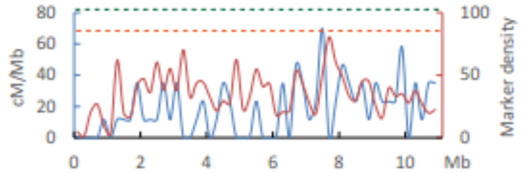

Chr 10

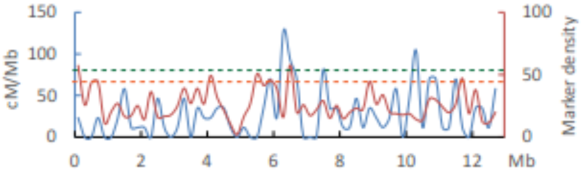

Chr 11

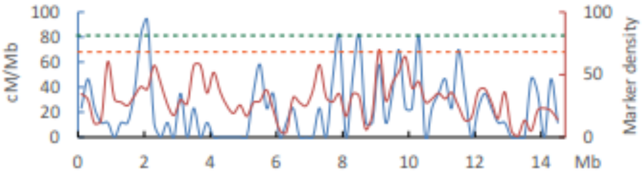

Chr 12

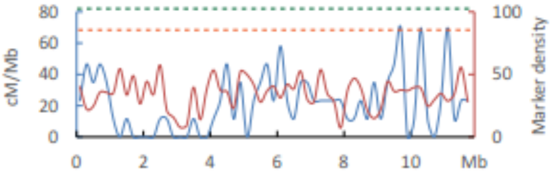

Chr 13

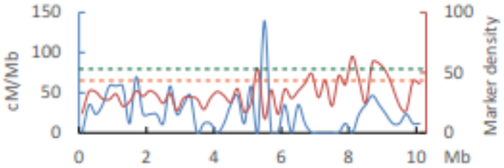

Chr 14

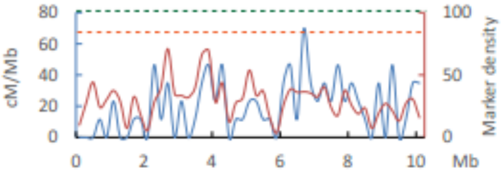

Chr 15

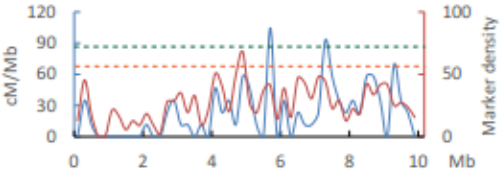

Chr 16

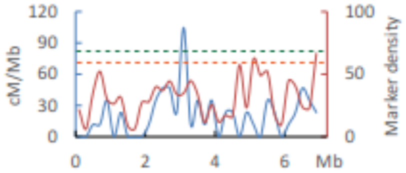

Figure S9. Plot of chromosome length against average number of crossovers or crossover rate on each chromosome. Blue (**A.**) red (**B.**) and green (**C.**) represent 10 kb tract, 100 kb tract and 500 kb sequence window tracts, respectively. **D.** Plot of chromosome length against crossover rate for 16 chromosomes. Crossover in 10 kb tract means that genotype change of tract length ≤10 kb are not counted as crossovers, and the same for crossover in 100 kb and 500 kb tracts.

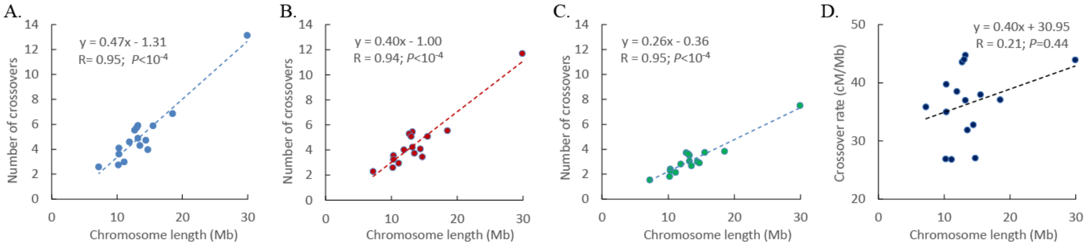

Figure S10. Relationship between recombination and GC-content. A. B. and **C.** are GC-content variation around CO breakpoints in colony I, II and III, respectively. Crossover events which can be delimited within a 2 kb interval and with no length-unknown gaps located within the interval are used to construct this figure. **D.** Distribution of GC-content within breakpoint intervals (red) compared with whole-genome GC-content distribution (blue). 2113 crossover breakpoints which can be delimited within a 10 kb interval and with no length-unknown gap located within the interval are used to construct the red line. The average length of these intervals is 1.4 kb. Then the whole genome is divided into continuous 1.4 kb long regions, GC-content of these regions being calculated to construct the blue line. **E.** The genome is dissected into 10 kb non-overlapping windows, then the windows are grouped based on GC-content (44 groups, 14%~58%, 1% interval), the figure shows the plot of average recombination rate against GC-content for the 44 groups. A great variance in recombination rate is found in the last 3 groups (GC-content: 55%~58%), the reason is these 3 groups only cover 40 kb of the genome. For example, the last groups covers 10 kb of the genome, and 3 crossover events in the 43 drones is identified is this region, which resulting in a very high recombination rate. After removing these 3 groups, the correlation improved significantly (**F.**). To get a reliable result, only groups with >100 windows are used to construct Figure 4B. Lines in E and F are major axes regression lines. Error bars = s.e.m.

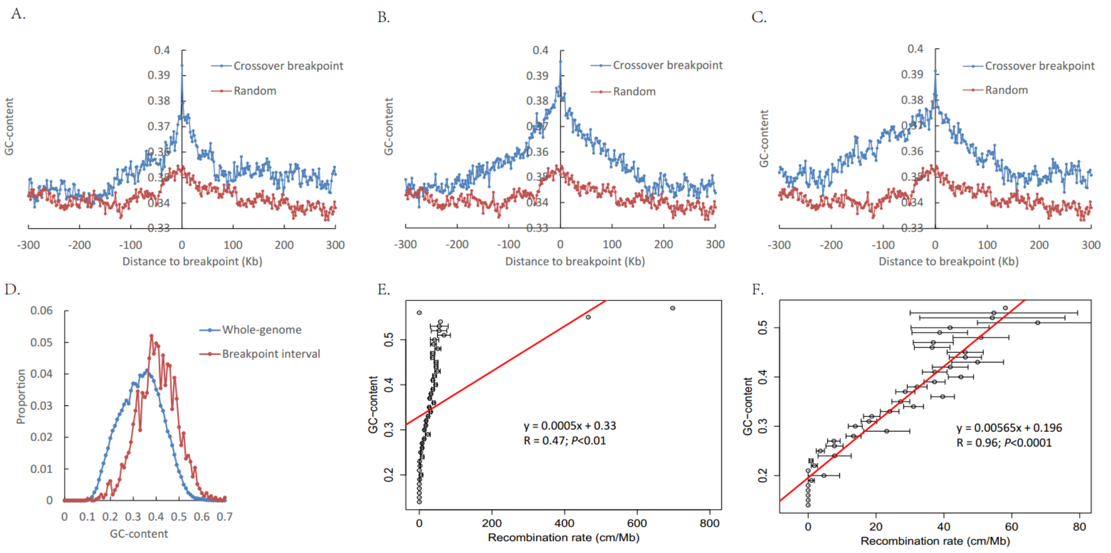

Figure S11. Plot of nucleotide diversity between the two haplotypes against recombination rate at different scales. A. B. C. and **D.** are constructed in non-overlapping 10 kb, 100 kb, 200 kb and 500 kb windows, respectively. When constructing in 10 kb windows, the genome was first dissected into non-overlapping 10 kb windows and then grouped by heterozygosity (0~0.01, 0.00025 interval). The average recombination rate and heterozygosity is obtained for each group, and groups with combined length > 1 Mb (i.e. > 100 windows in 10 kb) were used to construct Figure A, the same method is used for B, C and D. On average, no less than 97% of all windows were used in constructing these figures. Error bars = s.e.m. Spearman's rho values for all the windows unbinned are 0.132 for A ($P<10^{-15}$), 0.318 for B ($P<10^{-15}$), 0.236 for C ($P<10^{-14}$) and 0.242 for D ($P<10^{-16}$).

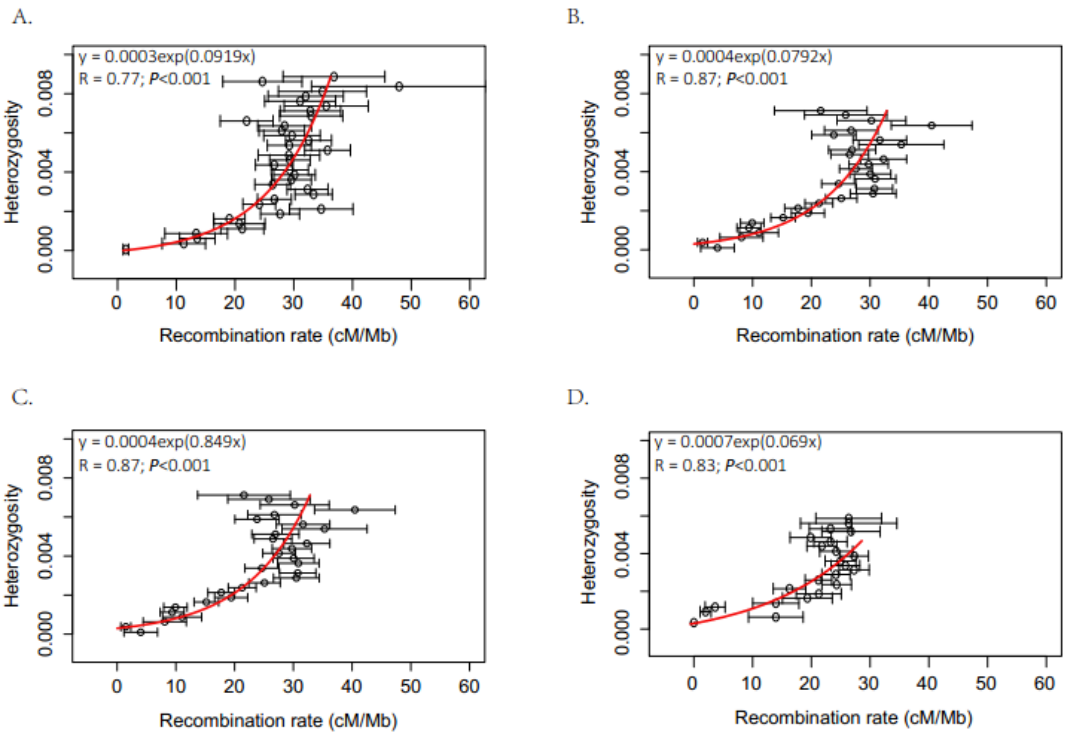

Figure S12. Plot of gene density against recombination rate in 100 kb non-overlapping sequence windows. The genome was dissected into non-overlapping 100 kb windows and then grouped by number of harbored genes (0~25, 1 interval). The average recombination rate and number of genes is obtained for each group, and groups with combined length > 1 Mb (i.e. > 10 windows in 100 kb) were used to construct this figure, 99.0% of all windows were used. Error bars = s.e.m.. Spearman's rho for all the windows unbinned is -0.141 ($P<10^{-10}$).

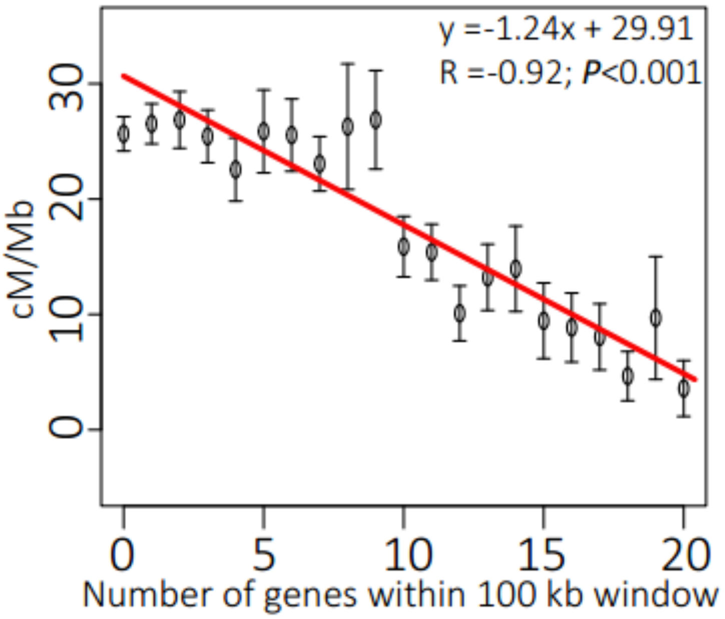

Figure S13. Plot of the length of multi-copy regions against recombination rate. A. B. and **C.** are constructed in 100 kb, 200 kb and 500 kb non-overlapping windows, respectively. When constructing in 100 kb windows, the genome was first dissected into non-overlapping 100 kb windows and then grouped by proportion of multi-copy region (0~50%, 2% interval). The average recombination rate and proportion of multi-copy region is obtained for each group, and groups with combined length > 1 Mb (i.e. > 10 windows in 100 kb) were used to construct Figure A, the same method is used for B and C. On average, no less than 98% of all windows were used in constructing these figures. Error bars = s.e.m. Spearman's rho for all the windows unbinned are 0.359 for A (P<10^{-15}), 0.383 for B (P<10^{-15}) and 0.397 for C (P<10^{-15}).

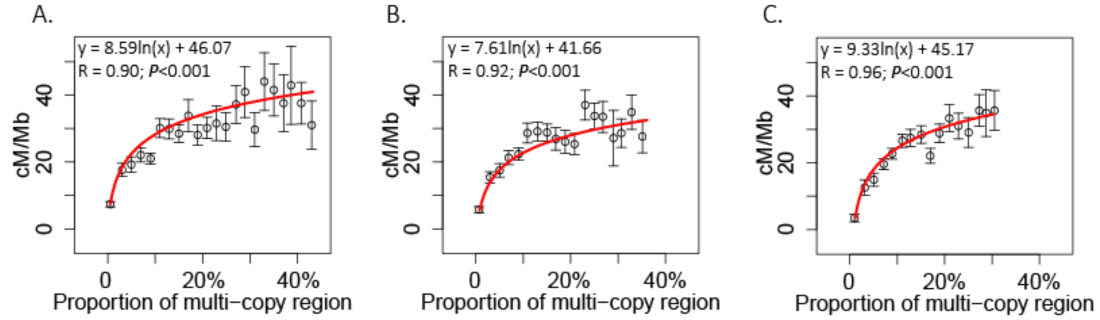

Figure S14. Recombination rate variation around gene *fem* and *csd*. Recombination rate is constructed using a 50 kb sliding window. Gene *fem* and *csd* are represented by the yellow and green bars, respectively.

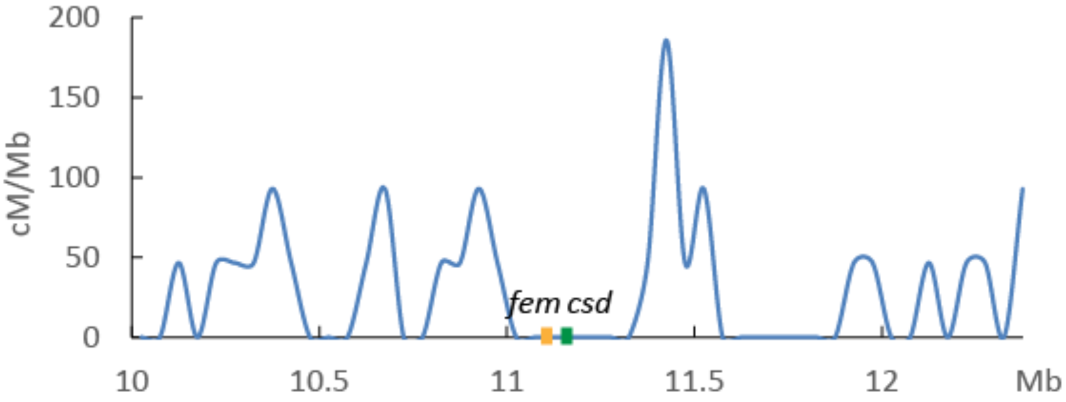

Figure S15. Schematic diagram of a site with 7 out of 15 crossovers. We show the genotypes of 15 drones on part of chromosome 1. In the first row, 'D' is short for 'Drone': 'D1' to 'D15' representing the 15 drones. Shared crossover events are found in between markers 'LG1 13015265' and 'LG1 13071669'. There are two possible original haplotypes between these two markers, 'A-A; T-G' (**A.** 8 original links against 7 recombined links) or 'A-G; T-A' (**B.** 7 original links against 8 recombined links). In cases like this, the majority are determined as original haplotypes, as shown in figure A. Nevertheless, a number of crossover events can clearly distinguished in a few drones within this 56405 bp region.

A.

	Original haplotypes								Recombined haplotypes						
Position	D1	D2	D3	D4	D5	D6	D7	D8	D9	D10	D11	D12	D13	D14	D15
LG1 13015014	C	C	C	C	C	A	A	A	A	A	A	A	C	C	C
LG1 13015023	G	G	G	G	G	A	A	A	A	A	A	A	G	G	G
LG1 13015030	T	T	T	T	T	C	C	C	C	C	C	C	T	T	T
LG1 13015228	T	T	T	T	T	C	C	C	C	C	C	C	T	T	T
LG1 13015265	A	A	A	A	A	T	T	T	T	T	T	T	A	A	A
LG1 13071669	A	A	A	A	A	G	G	G	A	A	A	A	G	G	G
LG1 13073256	A	A	A	A	A	G	G	G	A	A	A	A	G	G	G
LG1 13073548	C	C	C	C	C	T	T	T	C	C	C	C	T	T	T
LG1 13073827	G	G	G	G	G	A	A	A	G	G	G	G	A	A	A
LG1 13073877	G	G	G	G	G	A	A	A	G	G	G	G	A	A	A
LG1 13073933	A	A	A	A	A	G	G	G	A	A	A	A	G	G	G

B.

	Recombined haplotypes								Original haplotypes						
Position	D1	D2	D3	D4	D5	D6	D7	D8	D9	D10	D11	D12	D13	D14	D15
LG1 13015014	C	C	C	C	C	A	A	A	A	A	A	A	C	C	C
LG1 13015023	G	G	G	G	G	A	A	A	A	A	A	A	G	G	G
LG1 13015030	T	T	T	T	T	C	C	C	C	C	C	C	T	T	T
LG1 13015228	T	T	T	T	T	C	C	C	C	C	C	C	T	T	T
LG1 13015265	A	A	A	A	A	T	T	T	T	T	T	T	A	A	A
LG1 13071669	A	A	A	A	A	G	G	G	A	A	A	A	G	G	G
LG1 13073256	A	A	A	A	A	G	G	G	A	A	A	A	G	G	G
LG1 13073548	C	C	C	C	C	T	T	T	C	C	C	C	T	T	T
LG1 13073827	G	G	G	G	G	A	A	A	G	G	G	G	A	A	A
LG1 13073877	G	G	G	G	G	A	A	A	G	G	G	G	A	A	A
LG1 13073933	A	A	A	A	A	G	G	G	A	A	A	A	G	G	G

Figure S16. Decision flow of NCO and CO discrimination in this study.

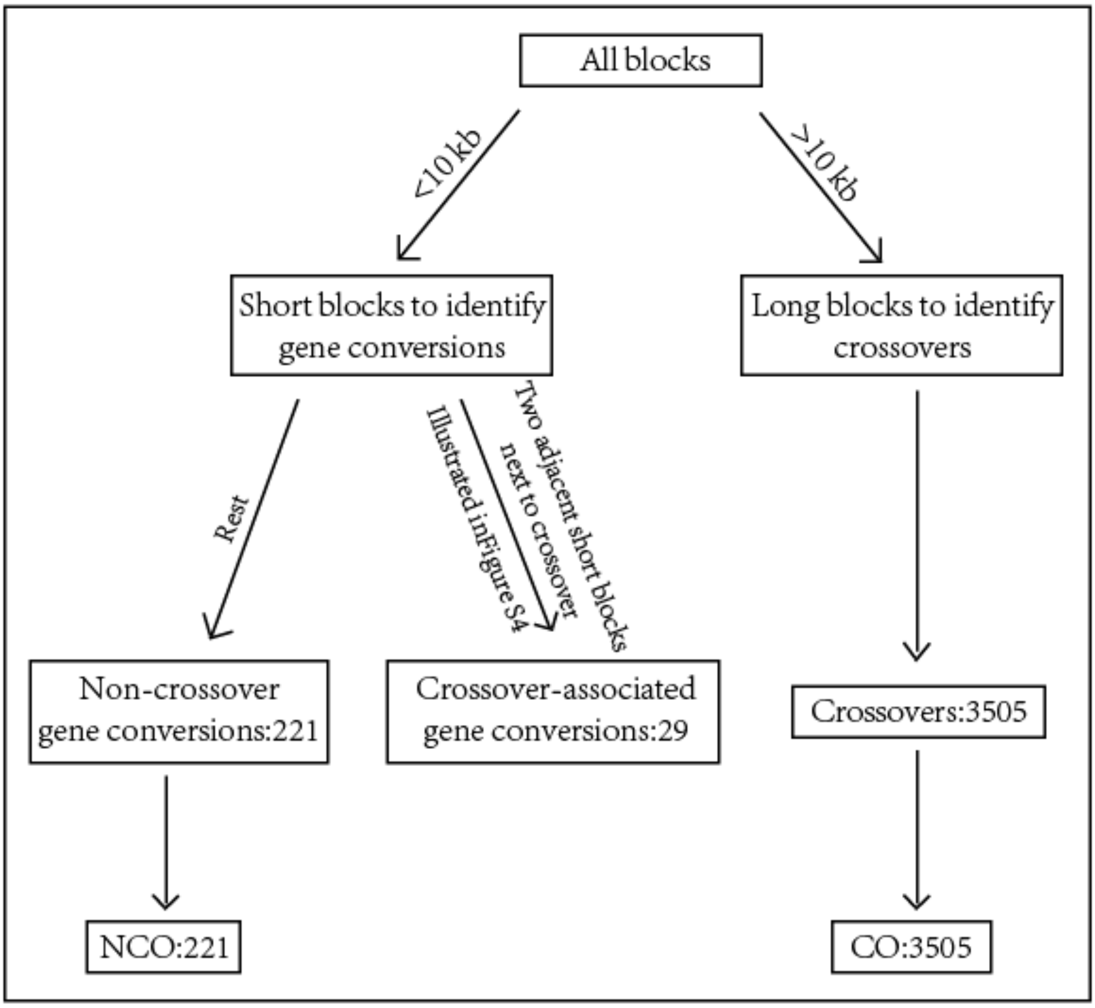

Figure S17. Depth distribution at drone-hetSNP sites (blue) and at marker sites (red) in drones.

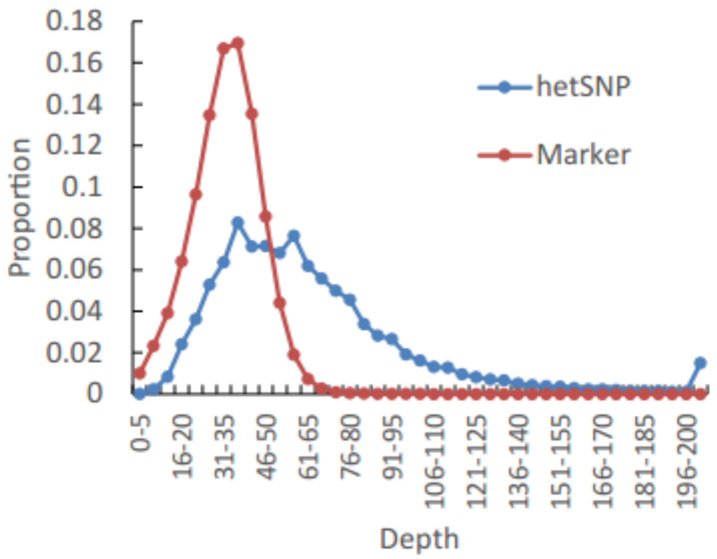

Supplement: Additional file 1: Figure S1. — Polygenetic relationship of the drones sequenced in this study, including the putative haplotypes of three queens. Figure S2. Schematic diagram of the process of haplotype phasing along the chromosome. Figure S3. Histogram of crossover frequency in drones for all crossover sites in the genome. Figure S4. Schematic diagram of crossover-associated gene conversion event. Figure S5. The effects of different thresholds for gene conversion and crossover on estimated rates. Figure S6. Plot of number of markers randomly picked against inferred recombination rate. Figure S7. Schematic diagram of gene conversion events in multi-copy region. Figure S8. Recombination rate and marker density variation along chromosomes 1 to 16. Figure S9. Plot of chromosome length against average number of crossovers or crossover rate on each chromosome. Figure S10. Relationship between recombination and GC content. Figure S11. Plot of nucleotide diversity between the two haplotypes against recombination rate at different scales. Figure S12. Plot of gene density against recombination rate in 100 kb non-overlapping sequence window. Figure S13. Plot of length of multi-copy region against recombination rate. Figure S14. Recombination rate variation around gene fem and csd. Figure S15. Schematic diagram of a site with seven out of 15 crossovers. Figure S16. Decision flow of NCO and CO discrimination in this study. Figure S17. Depth distribution at drone-hetSNP sites (blue) and at marker sites (red) in drones. [file 13059_2014_566_MOESM1_ESM.pdf]
