# Supplementary material for: Causes and consequences of crossing-over evidenced via a high-resolution recombinational landscape of the honey bee
Source: Genome Biol. 2015 Jan 2;16(1):15. doi: 10.1186/s13059-014-0566-0 (PMC4305242; doi:10.1186/s13059-014-0566-0)
Supplement: Additional file 2: Table S1. — Summary statistics for sequencing data. Table S2. Detected markers in three honeybee colonies. Table S3. HetSNPs detected in drones. Table S4. Number of crossovers and gene conversions in each sequenced sample. Table S5. Gene conversion events detected in multi-copy regions. Table S6. List of crossover hot- and cold-spot regions. Table S7. Statistics of CO hotspots in each colony. Table S8. Relationship between recombination regions or/and multi-copy regions and genes with biased expression in brain or fat body. Table S9. List of 17 genes related to nervous system or behavior. Table S10. DAVID annotation clusters enriched in genes overlap with crossover. [file 13059_2014_566_MOESM2_ESM.pdf]

Table S1. Summary statistics for sequencing data.

Colony A			Colony C			Colony E		
Sample	Genome Coverage	Mean Depth	Sample	Genome Coverage	Mean Depth	Sample	Genome Coverage	Mean Depth
Queen	98%	81.02	Queen	98%	59.73	Queen	98%	61.52
A8* (1st Seq.)	96%	26.33	C5	98%	34.51	ED3	97%	30.18
A8* (2nd Seq.)	90%	23.89	C6	98%	35.84	ED4	97%	29.95
A9	98%	48.32	C7	98%	35.06	ED5	98%	30.01
A17	97%	25.06	C8	98%	35.47	ED6	97%	29.89
A19	98%	23.58	C16	98%	35.76	ED8	97%	29.56
A20	95%	22.64	C17	98%	35.39	ED13	97%	29.78
A23	98%	50.41	C23	98%	35.52	ED16	98%	29.79
A38* (1st Seq.)	93%	30.58	C24	98%	35.17	ED17	98%	30.13
A38* (2nd Seq.)	84%	24.06	C26	98%	35.48	ED18	96%	30.22
A40	98%	45.76	C27	98%	35.12	ED21	95%	30.57
A41	98%	30.08	C28	98%	35.15	ED22	98%	30.10
A42	98%	73.62	C32	98%	37.88	ED28	97%	30.72
A48	98%	48.53	C35	98%	36.36	ED30	97%	30.00
A54	90%	35.35	C36	98%	35.54	EW2	97%	30.52
A58	95%	35.31	C38	98%	35.50	EW8	98%	30.57
A59	98%	80.36				EW9	98%	29.62
A61* (1st Seq.)	96%	35.5				EW16	98%	30.25
A61* (2nd Seq.)	94%	23.84				EW24	98%	30.94
						EW29	98%	30.27

*Three samples, A8, A38 and A61, were sequenced twice.

In the sample column, A8-A61, C5-C38 and ED3-ED30 are stand for drones and EW2-EW29 are stand for workers.

Table S2. Detected markers in three honeybee colonies.

		Colony A	Colony C	Colony E
HetSNPs detected in queen (Quality score>30)		960, 246 (100%)	954, 527 (100%)	920, 528 (100%)
Removed markers	HetSNP is called in 1 or more drones [a]	227, 975(24%)	197, 342 (21%)	206, 607 (22%)
	No coverage, deletion or low quality [b]	59, 514 (6%)	13, 580 (1%)	25, 577 (3%)
	Same genotype in all dornes or drones have different genotype from queen	823 (0.09%)	2, 605 (0.3%)	728 (0.08%)
	SNPs around indel [c]	244 (0.03%)	237 (0.02%)	152 (0.02%)
**Final markers used to detect recombination events**		671, 690 (70%)	740, 763 (78%)	687, 464 (75%)

[a] For hetSNP sites in each queen, if hetSNP also are called in 1 or more drones at this site, then this marker is removed due to potential multi-copy in this region.

[b] For each hetSNP site, if ≥ 1 drone have no coverage, deletion or low quality score (<30) at this site, then this marker is removed.

[c] For these markers around indels, some of them were found to be false due to the misalignment, and false markers will introduce genotype change. Therefore, in small genotype blocks (<10 Kb), all of the markers around indels within 20 bp were removed.

Table S3. HetSNPs detected in drones. Only these ≥2 linked hetSNPs in the drones were used to detect multi-copy regions in the genomes.

	Colony A	Colony C	Colony E
HetSNP is called in 1 or more drones	227, 975 (100%)	197, 342 (100%)	206, 607 (100%)
HetSNP is called in 2 or more drones	177, 671 (78%)	175, 731 (89%)	180, 460 (87%)
≥2 linked hetSNPs (Distance<2 kb)	169, 805 (74%)	167, 575 (85%)	172, 383 (83%)
No. of drone-hetSNPs sites in each colony	**169, 805**	**167, 575**	**172, 383**
Shared hetSNPs by 3 colonies	53, 989	53, 989	53, 989
Shared hetSNPs by 2 colonies only	57, 987	48, 899	52, 258
Specific hetSNPs	57, 829	64, 687	66, 136
No. of regions with hetSNPs (Length, Mb)	16984 (28.8)	16938 (30.7)	17141 (30.2)
Shared regions by 3 colonies (Mb)	9854 (22.5)	9691 (23.4)	9772 (23.3)
Shared by 2 colonies only (Mb)	4624 (4.5)	4145 (4.8)	4253 (4.6)
Colony Specify	2506 (1.8)	3102 (2.5)	3115 (2.3)

The combined length of 16 chromosomes is 219.63 Mb.

Table S4. Number of crossovers and gene conversions in each sequenced sample. These numbers include multiple crossovers between a given pair of markers.

Sample	Number of crossovers （≥10 kb）	Number of crossovers （≥100 kb）	Number of crossovers （≥500 kb）	Number of gene conversions
A8	88	80	51	5
A9	67	64	42	4
A17	86	74	44	10
A19	86	78	55	10
A20	73	66	52	9
A23	82	73	45	10
A38	97	83	55	8
A40	84	75	45	7
A41	79	72	49	9
A42	89	69	50	10
A48	90	76	51	1
A54	71	68	45	8
A58	78	71	44	5
A59	87	76	52	6
A61	74	64	47	7
C5	87	77	54	7
C6	103	85	53	7
C7	73	65	41	4
C8	91	81	56	3
C16	67	59	50	6
C17	85	72	45	5
C23	91	84	55	4
C24	91	77	59	1
C26	69	54	46	4
C27	80	67	50	8
C28	88	81	59	8
C32	85	74	61	6
C35	89	81	53	5
C36	89	77	55	4
C38	87	77	55	0
ED3	82	75	46	4
ED4	84	76	55	4
ED5	77	75	52	8
ED6	72	70	51	9
ED8	67	59	51	2
ED13	74	64	51	6
ED16	85	77	62	3
ED17	81	72	51	6

ED18	73	64	44	11
ED21	81	77	51	7
ED22	84	78	52	3
ED28	60	56	41	3
ED30	79	73	49	7
EW2	90	78	50	*
EW8	69	61	42	*
EW9	87	75	58	*
EW16	81	70	54	*
EW24	75	67	48	*
EW29	90	81	49	*

* The genomes of workers did not used to detect gene conversion due to high errors calling gene conversions in the heterozygous genome.

Table S5. Gene conversion events detected in multi-copy regions. The three types of gene conversion here (Het -> Hom, Hom -> Het and Het-Hom -> Het-Hom) corresponding to supplemental figure 6A, 6B and 6C, respectively.

**Type**	**Number of events**
Het -> Hom	14
Hom -> Het	16
Het-Hom -> Het-Hom	15

Table S6. List of crossover hot- and cold-spot regions. Defined by Poisson distribution with *P*<0.01 and *P*<0.05, respectively, window size is 200 Kb. These numbers do not include multiple crossovers between a given pair of markers.

Hot regions (Chromosome:Start-end, Mb)	Recombination rate (cM/Mb)	*P*-value
LG1:0.6-0.8	69.76744186	*P*<0.05
LG1:1.4-1.6	81.39534884	*P*<0.01
LG1:2.2-2.4	81.39534884	*P*<0.01
LG1:3.4-3.6	69.76744186	*P*<0.05
LG1:4-4.2	116.2790698	*P*<0.01
LG1:8.4-8.6	69.76744186	*P*<0.05
LG1:9.6-9.8	69.76744186	*P*<0.05
LG1:15.4-15.6	69.76744186	*P*<0.05
LG1:16.8-17	81.39534884	*P*<0.01
LG1:19.4-19.6	69.76744186	*P*<0.05
LG1:23.4-23.6	69.76744186	*P*<0.05
LG1:28.8-29	69.76744186	*P*<0.05
LG10:5.8-6	69.76744186	*P*<0.05
LG10:6.2-6.8	104.6511628	*P*<0.01
LG10:7.4-7.6	81.39534884	*P*<0.01
LG10:10.2-10.4	104.6511628	*P*<0.01
LG10:10.6-11	69.76744186	*P*<0.05
LG10:11.4-11.6	69.76744186	*P*<0.05
LG11:1.8-2.2	87.20930233	*P*<0.01
LG11:7.8-8	81.39534884	*P*<0.01
LG11:8.4-8.6	81.39534884	*P*<0.01
LG11:9.6-9.8	69.76744186	*P*<0.05
LG11:10.2-10.4	81.39534884	*P*<0.01
LG11:11.4-11.6	69.76744186	*P*<0.05
LG12:9.6-9.8	69.76744186	*P*<0.05
LG12:10.2-10.4	69.76744186	*P*<0.05

LG12:11-11.2	69.76744186	*P*<0.05
LG13:1.6-1.8	69.76744186	*P*<0.05
LG13:5.4-5.6	139.5348837	*P*<0.01
LG14:6.6-6.8	69.76744186	*P*<0.05
LG15:5.6-5.8	104.6511628	*P*<0.01
LG15:7.2-7.4	93.02325581	*P*<0.01
LG15:9.2-9.4	69.76744186	*P*<0.05
LG16:3-3.2	104.6511628	*P*<0.01
LG2:6.2-6.4	197.6744186	*P*<0.01
LG2:8.4-8.6	69.76744186	*P*<0.05
LG2:14-14.2	93.02325581	*P*<0.01
LG3:4.4-4.6	69.76744186	*P*<0.05
LG3:5.2-5.4	69.76744186	*P*<0.05
LG3:9.6-9.8	69.76744186	*P*<0.05
LG4:2.2-2.4	81.39534884	*P*<0.01
LG4:7.2-7.4	69.76744186	*P*<0.05
LG4:8.8-9	69.76744186	*P*<0.05
LG4:10.2-10.4	93.02325581	*P*<0.01
LG4:12-12.2	69.76744186	*P*<0.05
LG5:12.4-12.6	93.02325581	*P*<0.01
LG6:5.6-5.8	197.6744186	*P*<0.01
LG6:13.8-14	69.76744186	*P*<0.05
LG6:18-18.4	93.02325581	*P*<0.01
LG7:2.6-2.8	69.76744186	*P*<0.05
LG7:3.2-3.4	69.76744186	*P*<0.05
LG7:3.6-3.8	81.39534884	*P*<0.01
LG7:10.6-10.8	69.76744186	*P*<0.05
LG8:3-3.2	69.76744186	*P*<0.05
LG8:5.6-5.8	69.76744186	*P*<0.05
LG8:6-6.2	81.39534884	*P*<0.01
LG8:7.6-7.8	81.39534884	*P*<0.01
LG9:7.4-7.6	69.76744186	*P*<0.05

Cold regions (Chromosome:Start-end, Mb)	Recombination rate (cM/Mb)	*P*-value
LG1:5.8-6.4	0	*P*<0.01
LG1:6.6-7	0	*P*<0.01
LG1:11.8-12.8	0	*P*<0.01
LG1:14.6-15.2	0	*P*<0.01
LG1:19.8-20.2	0	*P*<0.05
LG1:21.0-21.4	0	*P*<0.05
LG2:0.2-0.6	0	*P*<0.05
LG2:0.8-2.0	0	*P*<0.01
LG2:3.2-3.6	0	*P*<0.05
LG4:0.4-1.0	0	*P*<0.01

LG4:1.2-1.6	0	$P<0.05$
LG4:4.6-5.0	0	$P<0.05$
LG4:11.0-11.4	0	$P<0.05$
LG5:0-0.4	0	$P<0.05$
LG5:3.2-3.6	0	$P<0.05$
LG5:13.4-13.8	0	$P<0.05$
LG6:0.8-1.2	0	$P<0.05$
LG6:2.2-2.8	0	$P<0.01$
LG6:4.4-4.8	0	$P<0.05$
LG6:6-6.4	0	$P<0.05$
LG6:10.6-11	0	$P<0.05$
LG6:13.2-13.6	0	$P<0.05$
LG6:16.6-17	0	$P<0.05$
LG6:17.2-17.6	0	$P<0.05$
LG7:4.6-5.2	0	$P<0.01$
LG7:6.2-7.8	0	$P<0.01$
LG7:8-8.4	0	$P<0.05$
LG8:0.2-0.6	0	$P<0.05$
LG8:1.6-2.6	0	$P<0.01$
LG8:6.2-6.8	0	$P<0.01$
LG8:8.8-9.2	0	$P<0.05$
LG8:12.2-13	0	$P<0.01$
LG9:0-0.8	0	$P<0.01$
LG9:3.2-3.6	0	$P<0.05$
LG9:4.8-5.4	0	$P<0.01$
LG9:5.6-6.2	0	$P<0.01$
LG10:0.2-0.6	0	$P<0.05$
LG10:0.8-1.2	0	$P<0.05$
LG10:5.2-5.6	0	$P<0.05$
LG10:6.8-7.4	0	$P<0.01$
LG11:4-5.2	0	$P<0.01$
LG11:6.6-7.2	0	$P<0.01$
LG11:13-13.6	0	$P<0.01$
LG12:1.6-2.4	0	$P<0.01$
LG12:2.8-3.4	0	$P<0.01$
LG12:3.6-4	0	$P<0.05$
LG13:5.6-6	0	$P<0.05$
LG13:6.8-7.8	0	$P<0.01$
LG14:0-0.6	0	$P<0.01$
LG14:1.2-1.6	0	$P<0.05$
LG15:0.6-2	0	$P<0.01$
LG15:2.2-2.6	0	$P<0.05$
LG16:0-0.4	0	$P<0.05$
LG16:1.4-2	0	$P<0.01$

Table S7. Statistics of CO hotspots in each colony. These numbers do not include multiple crossovers between a given pair of markers.

	Colony A		Colony C		Colony E	
	P<0.05	*P*<0.01	*P*<0.05	*P*<0.01	*P*<0.05	*P*<0.01
No. of CO hotspots (Length, Mb)	52 (10.8)	15 (3.0)	61 (13.0)	19 (4.2)	40 (8.0)	12 (2.4)
Shared by 3 colonies (Mb)	3 (0.6)	1 (0.2)	3 (0.6)	1 (0.2)	3 (0.6)	1 (0.2)
Shared by 2 colonies only (Mb)	9 (1.8)	2 (0.4)	9 (1.8)	3 (0.6)	8 (1.6)	3 (0.6)
Colony specified (Mb)	40 (8.4)	12 (2.4)	49 (10.6)	15 (3.4)	29 (5.8)	8 (1.6)
No. of COs within hotspots (%)	186 (25%)	69 (9%)	224 (29%)	92 (12%)	139 (22%)	52 (8%)

CO hotspots are defined by Poisson distribution with *P*<0.05 and *P*<0.01 based on whole genome CO distribution (Window size: 200 Kb).

Table S8. Relationship between recombination regions or/and multi-copy regions and genes with biased expression in brain or fat body. The statistics were performed by Chi-square test with a 2 by 2 table comparing between whole-genomic genes and the biased genes. All these results have shown that genes with worker-brain biased expression are strongly associated with the recombination (P <0.029 to 2.2×10^{-16}), whereas the drone-biased expression genes are significantly deviated from the regions with recombination in the honeybees (P <1.7×10^{-10} to 2.2×10^{-16}); in fat body, genes with drone-biased expression are strongly associated with the recombination (P <0.0002).

	Sets of genes	Genes within or around the breakpoints	Genes away from the breakpoints	Chi-square test with a 2 by 2 table, df.=1	
				χ^2	*P*
A. Brain expression genes within break-point regions or around the break-point regions within 10 kb	**Whole-genome genes**	**1745**	**10373**	*	*
	Worker-biased genes in reference (1)	70	312	4.77	**0.029**
	Worker-biased genes in reference (2)	128	317	74.49	**2.2e-16**
	Worker-biased expression genes overlapping references (1)and (2)	22	44	19.19	**1.2e-5**
	Union set of worker-biased expression genes in references (1) and (2)	173	579	45.18	**1.8e-11**
	Queen-biased genes in reference (1)	67	456	1.07	0.30
	Drone-biased genes in reference (2)	77	958	40.68	**1.7e-10**
	Positive selected genes in reference (3)	159	704	11.34	**7.6e-4**
B. Brain expression genes in multi-copy regions in our three colonies	**Whole-genome genes**	**2644**	**9474**	*	*
	Worker-biased genes in reference (1)	112	270	12.60	**3.9e-4**
	Worker-biased genes in reference (2)	186	259	104.13	**2.2e-16**
	Worker-biased expression genes overlapping references (1)and (2)	35	31	37.69	**8.3e-10**
	Union set of worker-biased expression genes in references (1) and (2)	263	489	76.29	**2.2e-16**
	Queen-biased genes in reference (1)	117	406	0.09	0.76
	Drone-biased genes in reference (2)	108	927	78.63	**2.2e-16**
	Positive selected genes in reference (3)	282	581	59.64	**1.1e-14**
C. Overlapped Brain expression genes within break-point regions or around the break-point regions within 10 kb and multi-copy regions	**Whole-genome genes**	**3400**	**8718**	*	*
	Worker-biased genes in reference (1)	133	249	8.65	**3.3e-3**
	Worker-biased genes in reference (2)	220	225	100.78	**2.2e-16**
	Worker-biased expression genes overlapping references (1)and (2)	39	27	31.49	**2.0e-8**
	Union set of worker-biased expression genes in references (1) and (2)	314	438	69.90	**2.2e-16**
	Queen-biased genes in reference (1)	141	382	0.31	0.58
	Drone-biased genes in reference (2)	158	877	83.90	**2.2e-16**
	Positive selected genes in reference (3)	340	523	54.98	**1.2e-13**
D. Fat body expression genes within break-point regions or around the break-point	**Whole-genome genes**	**1745**	**10373**	*	*
	Worker-biased genes in reference (4)	5	39	0.33	0.57
	Queen-biased genes in reference (4)	15	105	0.35	0.55
	Drone-biased genes in reference (4)	15	29	13.84	**0.0002**

regions within 10 kb

Reference:

1. Grozinger CM, Fan Y, Hoover SER, Winston ML: **Genome-wide analysis reveals differences in brain gene expression patterns associated with caste and reproductive status in honey bees (Apis mellifera).** *Mol Ecol* 2007, **16**: 4837–4848.

2. Zayed A, Naeger NL, Rodriguez-Zas SL, Robinson GE: **Common and novel transcriptional routes to behavioral maturation in worker and male honey bees.** *Genes Brain Behav* 2012, **11**: 253–261.

3. Harpur BA, Kent CF, Molodtsova D, Lebon J, Alqarni A, Owayss A, Zayed A: **Population genomics of the honey bee reveals strong signatures of positive selection on worker traits.** *Proc Natl Acad Sci* 2014, doi:10.1073/pnas.1315506111.

4. Chan QWT, Chan MY, Logan M, Fang Y, Higo H, Foster LJ: **Honey bee protein atlas at organ-level resolution**. *Genome Res* 2013, **23**: 1951–1960.

Table S9. List of 17 genes related to nervous system or behavior.

Gene Symbol	Function
AmE75	Involved in regulation of brain function [1, 2].
Cpx	Involved in neuronal communication and synaptic growth in fruit fly [3–5].
Ct	Regulate distinct patterns of dendrite branching [6].
Dh31-R1	Member of GPCR family 2, has hormone receptor activity.
Dl	Encodes neurogenic locus protein.
Mirr	Mediate convergent sensory neuron specification, local differentiation and topographic wiring in fruit fly [7].
NLGN3	Encodes neuronal cell surface protein.
NT8	Neurotransmitter transporter 8.
Par-1	Initiates neurodegeneration in fruit fly [8].
Pka-C3	Involved in progressive neurodegeneration [9].
Rgl	Regulates neuroblast cortical polarity and spindle orientation, and also study has showed that this gene may associated with aggressive behavior in fruit fly [10, 11].
Sema-2a	Affect axon steering, fasciculation, branching and synapse formation [12].
SK	Involved in courtship memory and mediate multiple forms of behavioral plasticity [13].
Ss	Necessary for the diversification of dendrite morphology of dendritic arborization neurons [14].
Toll-6	Functions as neurotrophin receptors in fruit fly [15].
Toll-10	In the same gene family with Toll-6.
Ubx	Plays a critical role in conferring segment-appropriate morphology and survival on individual neurons in the adult-specific ventral CNS [16].

References:

1. Paul RK, Takeuchi H, Matsuo Y, Kubo T: **Gene expression of ecdysteroid‐regulated gene E74 of the honeybee in ovary and brain**. *Insect Mol Biol* 2005, **14**:9–15.

2. Paul RK, Takeuchi H, Kubo T: **Expression of Two Ecdysteroid-Regulated Genes, *Broad-Complex* and *E75* , in the Brain and Ovary of the Honeybee (Apis mellifera L.)**. *Zoolog Sci* 2006, **23**:1085–1092.

3. Huntwork S, Littleton JT: **A complexin fusion clamp regulates spontaneous neurotransmitter release and synaptic growth**. *Nat Neurosci* 2007, **10**:1235–1237.

4. Xue M, Lin YQ, Pan H, Reim K, Deng H, Bellen HJ, Rosenmund C: **Tilting the balance between facilitatory and inhibitory functions of mammalian and Drosophila Complexins orchestrates synaptic vesicle exocytosis**. *Neuron* 2009,

64:367–380.

5. Jorquera RA, Huntwork-Rodriguez S, Akbergenova Y, Cho RW, Littleton JT: **Complexin controls spontaneous and evoked neurotransmitter release by regulating the timing and properties of synaptotagmin activity**. *J Neurosci Off J Soc Neurosci* 2012, **32**:18234–18245.

6. Grueber WB, Jan LY, Jan YN: **Different Levels of the Homeodomain Protein Cut Regulate Distinct Dendrite Branching Patterns of Drosophila Multidendritic Neurons**. *Cell* 2003, **112**:805–818.

7. Karim MR, Moore AW: **Convergent local identity and topographic projection of sensory neurons**. *J Neurosci Off J Soc Neurosci* 2011, **31**:17017–17027.

8. Nishimura I, Yang Y, Lu B: **PAR-1 Kinase Plays an Initiator Role in a Temporally Ordered Phosphorylation Process that Confers Tau Toxicity in Drosophila**. *Cell* 2004, **116**:671–682.

9. Bettencourt da Cruz A, Wentzell J, Kretzschmar D: **Swiss Cheese, a protein involved in progressive neurodegeneration, acts as a noncanonical regulatory subunit for PKA-C3**. *J Neurosci Off J Soc Neurosci* 2008, **28**:10885–10892.

10. Carmena A, Makarova A, Speicher S: **The Rap1-Rgl-Ral signaling network regulates neuroblast cortical polarity and spindle orientation**. *J Cell Biol* 2011, **195**:553–562.

11. Edwards AC, Mackay TFC: **Quantitative Trait Loci for Aggressive Behavior in Drosophila melanogaster**. *Genetics* 2009, **182**:889–897.

12. Goodman CS, Kolodkin AL, Luo Y, Püschel AW, Raper JA: **Unified Nomenclature for the Semaphorins/Collapsins**. *Cell* 1999, **97**:551–552.

13. Abou Tayoun AN, Pikielny C, Dolph PJ: **Roles of the Drosophila SK channel (dSK) in courtship memory**. *PloS One* 2012, **7**:e34665.

14. Kim MD, Jan LY, Jan YN: **The bHLH-PAS protein Spineless is necessary for the diversification of dendrite morphology of Drosophila dendritic arborization neurons**. *Genes Dev* 2006, **20**:2806–2819.

15. McIlroy G, Foldi I, Aurikko J, Wentzell JS, Lim MA, Fenton JC, Gay NJ, Hidalgo A: **Toll-6 and Toll-7 function as neurotrophin receptors in the Drosophila melanogaster CNS**. *Nat Neurosci* 2013, **16**:1248–1256.

16. Marin EC, Dry KE, Alaimo DR, Rudd KT, Cillo AR, Clenshaw ME, Negre N, White KP, Truman JW: **Ultrabithorax confers spatial identity in a context-specific manner in the Drosophila postembryonic ventral nervous system**. *Neural Develop* 2012, **7**:31.

Table S10. DAVID annotation clusters enriched in genes overlap with crossover. 4 clusters of genes showed functional enrichment in this gene set. Notably, the first cluster of genes (GPCRs), which is responsible for sensing signals and responding to environment, is enriched in genes that overlap with crossovers.

**Annotation Cluster 1**	Enrichment Score: 1.255062533368358			
**Category**	**Term**	**Count**	**%**	**PValue**
KEGG_PATHWAY	ame04080:Neuroactive ligand-receptor interaction	5	0.3004808	0.0012232
GOTERM_CC_FAT	GO:0031224~intrinsic to membrane	14	0.8413462	0.0014646
GOTERM_CC_FAT	GO:0016021~integral to membrane	13	0.78125	0.0127157
SP_PIR_KEYWORDS	receptor	11	0.6610577	0.0325653
SP_PIR_KEYWORDS	transmembrane	14	0.8413462	0.1407212
INTERPRO	IPR000276:7TM GPCR, rhodopsin-like	5	0.3004808	0.1915298
INTERPRO	IPR017452:GPCR, rhodopsin-like superfamily	5	0.3004808	0.1915298
SP_PIR_KEYWORDS	g-protein coupled receptor	6	0.3605769	0.1932541
SP_PIR_KEYWORDS	transducer	6	0.3605769	0.1932541
GOTERM_BP_FAT	GO:0007166~cell surface receptor linked signal transduction	6	0.3605769	0.3307096
GOTERM_BP_FAT	GO:0007186~G-protein coupled receptor protein signaling pathway	6	0.3605769	0.3307096

**Annotation Cluster 2**	Enrichment Score: 1.0521597001950618			
**Category**	**Term**	**Count**	**%**	**PValue**
COG_ONTOLOGY	Lipid metabolism	4	0.2403846	0.0225421
INTERPRO	IPR002018:Carboxylesterase, type B	3	0.1802885	0.175899
INTERPRO	IPR019819:Carboxylesterase type B, conserved site	3	0.1802885	0.175899

**Annotation Cluster 3**	Enrichment Score: 0.8548117966482504			
**Category**	**Term**	**Count**	**%**	**PValue**
GOTERM_MF_FAT	GO:0043565~sequence-specific DNA binding	6	0.3605769	0.0080733
GOTERM_MF_FAT	GO:0003677~DNA bingding	7	0.4206731	0.0117495
GOTERM_MF_FAT	GO:0003700~transcription factor activty	5	0.3004808	0.0562203
GOTERM_BP_FAT	GO:0006355~regulation of	6	0.3605769	0.0806785

	transcription DNA-dependent			
GOTERM_BP_FAT	GO:0051252~regulation of RNA metabolic process	6	0.3605769	0.0806785
GOTERM_MF_FAT	GO:0030528~transcription regulator activity	5	0.3004808	0.0851108
GOTERM_BP_FAT	GO:0006350~transcription	3	0.1802885	0.1041926
SP_PIR_KEYWORDS	dna-binding	6	0.3605769	0.1140531
GOTERM_MF_FAT	GO:0008270~zinc ion binding	4	0.2403846	0.1176367
GOTERM_BP_FAT	GO:0045449~regulation of transcription	6	0.3605769	0.1227442
SP_PIR_KEYWORDS	Transcription	3	0.1802885	0.1663828
SP_PIR_KEYWORDS	transcription regulation	4	0.2403846	0.1800322
SP_PIR_KEYWORDS	nucleus	5	0.3004808	0.3211783
SP_PIR_KEYWORDS	zinc	3	0.1802885	0.349871
GOTERM_MF_FAT	GO:0046872~metal ion binding	5	0.3004808	0.3632676
GOTERM_MF_FAT	GO:0043169~cation binding	5	0.3004808	0.4722642
GOTERM_MF_FAT	GO:0043167~ion binding	5	0.3004808	0.4722642
GOTERM_MF_FAT	GO:0046914~transition metal ion binding	4	0.2403846	0.5387913
SP_PIR_KEYWORDS	metal-binding	3	0.1802885	0.7700051

**Annotation Cluster 4**	Enrichment Score: 0.7484154350876936			
**Category**	**Term**	**Count**	**%**	**PValue**
GOTERM_CC_FAT	GO:0031224~intrinsic to membrane	14	0.8413462	0.0014646
GOTERM_CC_FAT	GO:0016021~integral to membrane	13	0.78125	0.0127157
PIR_SUPERFAMILY	PIRSF002443:acetylcholine receptor	4	0.2403846	0.035776
GOTERM_MF_FAT	GO:0005216~ion channel activity	5	0.3004808	0.0851108
GOTERM_MF_FAT	GO:0015267~channel activity	5	0.3004808	0.0851108
GOTERM_MF_FAT	GO:0022803~passive transmembrane transporter activity	5	0.3004808	0.0851108
GOTERM_MF_FAT	GO:0022838~substrate specific channel activity	5	0.3004808	0.0851108
SP_PIR_KEYWORDS	ionic channel	6	0.3605769	0.0921943
GOTERM_MF_FAT	GO:0042165~neurotransmitter binding	5	0.3004808	0.1019566
GOTERM_MF_FAT	GO:0030594~neurotransmitter receptor activity	5	0.3004808	0.1019566
SP_PIR_KEYWORDS	transmembrane	14	0.8413462	0.1407212
SP_PIR_KEYWORDS	ion transport	6	0.3605769	0.164769
SP_PIR_KEYWORDS	cell membrane	5	0.3004808	0.2092028
GOTERM_MF_FAT	GO:0005230~extracellular ligand-gated ion channel activity	4	0.2403846	0.2275406

GOTERM_MF_FAT	GO:0015276~ligand-gated ion channel activity	4	0.2403846	0.2275406
GOTERM_MF_FAT	GO:0022836~gated channel activity	4	0.2403846	0.2275406
GOTERM_MF_FAT	GO:0022834~ligand-gated channel activity	4	0.2403846	0.2275406
INTERPRO	IPR006029:Neurotransmitter-gated ion-channel transmembrane region	4	0.2403846	0.2301239
INTERPRO	IPR006201:Neurotransmitter-gated ion-channel	4	0.2403846	0.3066394
INTERPRO	IPR006202:Neurotransmitter-gated ion-channel ligand-binding	4	0.2403846	0.3066394
GOTERM_CC_FAT	GO:0045211~postsynaptic membrane	4	0.2403846	0.3133799
GOTERM_CC_FAT	GO:0044456~synapse part	4	0.2403846	0.3521745
GOTERM_CC_FAT	GO:0045202~synapse	4	0.2403846	0.3521745
SP_PIR_KEYWORDS	cell junction	4	0.2403846	0.3600918
SP_PIR_KEYWORDS	synapse	4	0.2403846	0.3600918
SP_PIR_KEYWORDS	postsynaptic cell membrane	4	0.2403846	0.3600918
GOTERM_CC_FAT	GO:0005886~plasma membrane	4	0.2403846	0.3911454
GOTERM_BP_FAT	GO:0006811~ion transport	5	0.3004808	0.4154792
INTERPRO	IPR018000:Neurotransmitter-gated ion-channel, conserved site	3	0.1802885	0.479224
SP_PIR_KEYWORDS	transport	7	0.4206731	0.5119118
GOTERM_CC_FAT	GO:0030054~cell junction	3	0.1802885	0.5463093
GOTERM_CC_FAT	GO:0044459~plasma membrane part	3	0.1802885	0.6272634
SP_PIR_KEYWORDS	membrane	6	0.3605769	0.8581477
